# Dual-Transcriptome Dissection of the Mechanisms Underlying Alfalfa Phenotypic Differences Induced by Two Rhizobial Isolates

**DOI:** 10.3390/microorganisms14030571

**Published:** 2026-03-03

**Authors:** Jian Guan, Weizhen Li, Jinli Li, Baofu Lu, Yilin Han, Yuan-Yuan Du, Xiaoyu Xu, Bingsen Zhao, Xilin Xie, Wen-Juan Kang, Shang-Li Shi

**Affiliations:** 1 Key Laboratory of Grassland Ecosystem, Gansu Agricultural University, Ministry of Education, Lanzhou 730070, China; guanj@st.gsau.edu.cn (J.G.); lubf@st.gsau.edu.cn (B.L.); hanyl@st.gsau.edu.cn (Y.H.); duyy@st.gsau.edu.cn (Y.-Y.D.); 1073323120083@st.gsau.edu.cn (X.X.); 1073323020047@st.gsau.edu.cn (B.Z.); xiexl@st.gsau.edu.cn (X.X.); 2 Pratacultural College, Gansu Agricultural University, Lanzhou 730070, China; 3 Pratacultural College, Xinjiang Agricultural University, Urumqi 830052, China; johnnie0918@xjau.edu.cn; 4 State Key Laboratory of Aridland Crop Science, Gansu Agricultural University, Lanzhou 730070, China; 1073323120596@st.gsau.edu.cn; 5 College Agronomy, Gansu Agricultural University, Lanzhou 730070, China

**Keywords:** symbiotic nitrogen fixation, rhizobial strains with contrasting nitrogen fixation efficiencies, dual RNA sequencing, microaerobic homeostasis, environmental adaptation

## Abstract

Different rhizobial strains can lead to distinct symbiotic phenotypes in alfalfa, yet molecular differences at the mature nodule stage remain unclear. Here, we analyzed 21-day post-inoculation (dpi) nodules induced by strains *WE2* and *WWL2*. We measured nitrogenase activity (acetylene reduction assay, ARA) and performed dual RNA-seq to compare gene expression in both the alfalfa host and the rhizobia. On the host side, *WE2*-induced nodules showed higher expression of mature nodule marker genes (*ENOD93* and leghemoglobin (Lb) genes) and higher expression of genes encoding SWEET transporters and amino acid and peptide transporters. Host differentially expressed genes were enriched in pathways related to transmembrane transport, redox and heme-related functions, and processes linked to maintaining microaerobic conditions. On the rhizobial side, *WE2* nodules showed higher expression of genes involved in microaerobic respiration and nitrogen fixation (e.g., *nif*/*fix* and key respiratory chain genes), whereas *WWL2* nodules showed higher expression of genes linked to transport, chemotaxis/motility, and environmental information processing. Together, these host and rhizobia expression patterns suggest coordinated differences between host pathways related to resource supply and microaerobic conditions and rhizobial expression programs for respiration and nitrogen fixation. Based on these associations, we propose a working model and provide candidate genes and pathways for functional validation and inoculant screening.

## 1. Introduction

Legume rhizobium symbiosis supports symbiotic nitrogen fixation, converting atmospheric dinitrogen into ammonium within root nodules and providing a major biological nitrogen input to agriculture [1,2]. Oldroyd and Downie described nodule development as a staged process that links signal exchange and infection with nodule organogenesis and the transition to a functioning nitrogen fixing organ [3]. Gao et al. reviewed how rhizobia traverse the epidermis and cortex through infection threads to reach developing nodule cells [4]. In alfalfa (*Medicago sativa*) production, the choice of *Sinorhizobium meliloti* inoculant can influence plant nitrogen status and fresh weight above ground, making it important to understand how strain differences translate into symbiotic phenotypes [5,6].

Rhizobial strains vary widely in their ability to colonize roots, support nodule development, and deliver effective nitrogen fixation [1,6]. D’Amours et al. compared multiple *S. meliloti* strains on alfalfa and reported pronounced differences in shoot regrowth and nodule traits after freezing stress, highlighting that symbiotic performance can diverge strongly among strains [5]. Poole et al. emphasized that differences in rhizobial genomes and regulatory programs can shift the balance between competitive fitness and nitrogen fixing output in the nodule environment [6].

Once nodules mature, efficient symbiosis requires tight coordination between host and bacteria while the plant simultaneously restricts pathogen-like outcomes [7]. Udvardi and Poole detailed how carbon, nitrogen, and other nutrients are exchanged across plant and bacterial membranes through specialized transport systems that underpin metabolic integration in nodules [8]. Jiang et al. showed that NIN-like transcription factors regulate leghemoglobin gene expression in nodules, linking host transcriptional control to establishment of the low-oxygen conditions required for nitrogenase activity [9]. Minguillón et al. further demonstrated that leghemoglobins support symbiotic nitrogen fixation by scavenging free heme and mitigating redox stress in nodules [10]. Van de Velde et al. identified nodule-specific cysteine-rich peptides that drive terminal differentiation of *S. meliloti* in *Medicago truncatula*, illustrating how host signals reshape bacterial physiology in planta [11]. Lin et al. reviewed how plant hormones intersect with symbiotic signaling to coordinate infection and nodule organogenesis and to tune nodule development [12]. Together, these studies indicate that strain-specific symbiotic outcomes at the mature nodule stage can involve coordinated variation in host resource supply, oxygen and redox homeostasis, and bacterial programs for respiration, nitrogen fixation, and environmental adaptation [1,6,8,10].

Despite major advances in early recognition and infection, the host and strain coupled regulatory logic that sustains mature nodule function remains less resolved, particularly for pathways that connect substrate exchange with oxygen and redox control [1,8,10]. Addressing this gap is important for mechanistic interpretation of strain effects and for evidence-based selection of high-performance inoculants [5,6].

Here, we compared two *S. meliloti* strains, *WWL2* and *WE2*, that reproducibly generate distinct symbiotic outcomes in alfalfa, combining plant growth measurements and nitrogenase activity assays with transcriptome profiling of 21 days post-inoculation nodules. Dual RNA sequencing enables quantification of host and bacterial gene expression from the same biological sample, allowing direct comparisons of coordinated responses under different inoculation conditions [13,14]. Dual transcriptomic analyses have been applied to legume nodules to resolve how plant and rhizobium gene expression varies across genotypes and developmental stages [15]. Using this framework, we ask whether phenotypic differences between strains at the mature nodule stage are accompanied by changes in host pathways related to carbon and nitrogen substrate supply, transport, and low-oxygen homeostasis, and whether these changes align with bacterial expression of microaerobic respiration and nitrogen fixation modules and with other adaptive processes. By integrating both sides of the interaction, we present a testable working model and a set of candidate genes and pathways for downstream functional validation and inoculant screening.

## 2. Materials and Methods

### 2.1. Plant Material and Rhizobial Inoculation

A nutrient solution sand culture system was used. The substrate consisted of river sand passed through a 30 mesh sieve and perlite mixed at 1:3 (*v*/*v*), and both substrates were sterilized prior to use [16]. Plants were supplied with a self-prepared nitrogen-free nutrient solution modified from Hoagland solution by omitting all nitrogen sources, and any required elements were provided using nitrogen-free salts [17]. When seedlings reached a uniform growth stage and had developed the first true leaf, inoculation was performed by root drenching with 1 mL bacterial suspension (OD_600_ = 0.5) per seedling, using strain *WWL2* or *WE2*. Uninoculated controls were not included because both isolates are plant growth promoting strains and the objective was to compare their relative symbiotic performance, including nitrogen fixation, under identical nitrogen-free conditions. Growth conditions were 28 °C with 14 h light and 20 °C with 10 h darkness, relative humidity of 60% to 70%, and a light intensity of 260 to 350 μmol·m^−2^·s^−1^. Nodules were harvested at 21 days post-inoculation for RNA extraction and dual transcriptome sequencing. For each inoculation treatment, three independent biological replicates were established as separate culture boxes, and each biological replicate contained 15 seedlings; nodules harvested at 21 dpi from each replicate were processed independently for downstream analyses. Strain sources and accession information are as follows: *WWL2* and *WE2* are isolated *S. meliloti* strains preserved in the Key Laboratory of Grassland Agro ecosystems, Ministry of Education, Gansu Agricultural University; accession numbers: *WE2*, MG575945.1; *WWL2*, MG575932.1.

### 2.2. RNA Extraction, Library Preparation, and Sequencing

Total RNA was extracted from nodule tissues using TRIzol™ Reagent (Thermo Fisher Scientific, Waltham, MA, USA). Residual genomic DNA was subsequently removed with RNase-free DNase I (Thermo Fisher Scientific, Waltham, MA, USA). RNA integrity was verified via 1% agarose gel electrophoresis and the RNA integrity number (RIN) was determined using an Agilent 2100 Bioanalyzer (Agilent Technologies, Santa Clara, CA, USA). Purity (OD_260/280_ and OD_260/230_) and concentration were assessed with a NanoPhotometer (IMPLEN GmbH, Munich, Germany), followed by accurate quantification using a Qubit 2.0 Fluorometer (Thermo Fisher Scientific, Waltham, MA, USA).

For each library, total RNA ≥ 2 μg, concentration ≥ 100 ng/μL, and OD_260/280_ between 1.8 and 2.2 were required.

To obtain transcript abundance profiles for the host and the symbiont separately, total RNA from the same sample was split into two aliquots for independent library construction. For the host-side library, TruSeq RNA Sample Preparation Kit v2 (Illumina, San Diego, CA, USA) was used: poly(A)+ mRNA was enriched with Oligo(dT) magnetic beads, fragmented by ultrasonication, converted to double-stranded cDNA, end-repaired, A-tailed, adapter-ligated, and PCR-amplified. For the symbiont-side library, rRNA was removed using the Ribo-Zero™ rRNA Removal Kit (Illumina, San Diego, CA, USA), followed by strand-specific library construction: dUTP was substituted for dTTP during second-strand synthesis, and the second strand was digested by UNG before PCR amplification to retain strand specificity. Libraries were quantified with PicoGreen (Quant-iT PicoGreen dsDNA Assay Kit, Thermo Fisher Scientific, Waltham, MA, USA) using a TBS-380 Mini-Fluorometer (Turner BioSystems, Sunnyvale, CA, USA), pooled at equimolar ratios, and sequenced on an Illumina platform in paired-end mode (2 × 150 bp). Each sample yielded ~2.5 Gb of data.

### 2.3. Quality Control, Alignment, and Expression Quantification

Raw sequencing reads were first subjected to quality control and filtering. Trimmomatic (v0.39) was used to remove adapters and low-quality sequences [18]: adapter sequences were trimmed; reads without insert fragments due to adapter self-ligation were removed; bases with a 3′-end quality score < 20 were trimmed, and reads were discarded if any base with quality < 10 remained after trimming; reads with >10% ambiguous bases (N) were removed; and reads shorter than 75 bp after trimming were discarded. The remaining reads were designated as clean reads. Host-side clean reads were aligned to the alfalfa reference genome and annotation files using HISAT2 (v2.2.1) [19] (reference genome version/accession and annotation sources as provided). Symbiont-side clean reads were aligned using Rockhopper (v2.0.3) [20] to the reference genome of the model strain *Sinorhizobium meliloti 1021* (RefSeq: GCF_000006965.1), which served as the backbone for rhizobial transcript alignment and functional annotation. Gene-level read-count matrices for both the host and the rhizobium were generated from alignments combined with corresponding annotation files for downstream differential expression analyses (alfalfa: HISAT2 v2.2.1; featureCounts/Subread v2.0.3 [21]; rhizobium: Rockhopper v2.0.3).

Nodule samples contain mixed total RNA from host cells and symbiotic rhizobia, with host transcripts dominating total RNA. Therefore, the rhizobial mapped rate (%) was defined as Mapped reads/total clean reads × 100% to reflect the proportion of bacterial signal in total reads, and it should not be directly compared with mapping rates from single-species transcriptomes. In this study, the rhizobial mapped rate ranged from 1.64% to 5.04%, corresponding to ~0.94–3.45 million mapped reads per sample (Supplementary Table S1).

### 2.4. Differential Expression Analysis

To reduce false positives caused by low read counts in the rhizobial libraries, we filtered out lowly expressed rhizobial genes using edgeR::filterByExpr [22]. This function applies a CPM threshold that is adjusted for library size and group size. We then performed differential expression analysis on raw counts using TMM normalization and controlled the false discovery rate.

Differential expression analysis was performed on the raw gene-count matrix using edgeR (Bioconductor, v4.8.2) with a negative binomial model [22]; differences in library size were corrected by trimmed mean of M-values (TMM) normalization [23]. To reduce false positives caused by low-count noise, lowly expressed genes were filtered prior to analysis using the default strategy of edgeR::filterByExpr. Differentially expressed genes (DEGs) were defined using FDR < 0.05 and |log_2_FC| ≥ 1. Sample reproducibility was evaluated by principal component analysis (PCA).

### 2.5. Functional Annotation and Enrichment Analysis

For enrichment analyses, significance was determined using FDR adjusted for multiple testing. Gene Ontology (GO) [24] and Kyoto Encyclopedia of Genes and Genomes (KEGG) [25] enrichment analyses used FDR < 0.05 as the significance threshold.

### 2.6. Modular Integration and Candidate Gene Selection

To integrate evidence from both the host and the symbiont and to focus on verifiable key nodes, candidate genes were screened following a “pathway-node-evidence” strategy: we used mature nodule functional markers and pathways related to substrate exchange and microaerobic homeostasis as the main line; combined differential expression magnitude with functional annotation to identify key transport/metabolic nodes; and, on the basis of statistical significance, compiled candidate gene lists. In the Section 4, we further propose testable mechanistic hypotheses in the form of a conceptual framework (working hypothesis), providing candidate gene sets for expression validation and functional verification.

### 2.7. Module Score Calculation

To quantify the differential trend of multi-gene changes within a functional module as a single metric, module scores were computed based on predefined gene sets for each functional module (Supplementary Table S2). Briefly, expression values for each gene in each sample were transformed by log_2_(x + 1) and then, for each gene, row-wise standardization across the six samples was performed (z-score), i.e., z_ij = (x_ij − mean_i)/sd_i, and finally, the z values of all genes within the same module were averaged within each sample to obtain the module score, Score_j = mean_i(z_ij). Here, x_ij denotes the expression level (FPKM) of gene i in sample j (after log_2_(x + 1) transformation for standardization). Differential expression analysis was performed on raw counts; FPKM/RPKM values were used only for visualization and descriptive scoring. This scoring scheme is intended as a descriptive gene-set summary and is conceptually related to single-sample gene-set scoring approaches (e.g., ssGSEA/GSVA) [26,27], with per-gene z-scoring used to mitigate scale differences among genes. Therefore, module scores should be interpreted as relative expression tendencies rather than direct measures of causal “investment”. A module score > 0 indicates higher overall activity of that module in the sample, whereas < 0 indicates lower activity. Coupling between cross-partner modules was assessed using Pearson correlation analysis (*n* = 6). Module score validity was assessed using independent measurements obtained from the same biological replicates, including shoot fresh weight, nitrogenase activity measured by the acetylene reduction assay, and RT qPCR measurements of representative genes from the corresponding modules.

### 2.8. Alfalfa Phenotyping and Nitrogenase Activity Assay

Harvested plants were cut at the crown to separate shoots and roots. For each sample, shoot tissues from 15 plants were pooled and weighed immediately, and the pooled fresh weight was recorded. Shoot fresh weight measurements were performed with independent biological replicates (*n* = 3), with 15 plants per biological replicate.

Nitrogenase activity was determined using the acetylene reduction assay (ARA) [28]. Nitrogenase activity measurements were performed with independent biological replicates (*n* = 3). For each biological replicate, nodules from the 15 plants were pooled, gently blotted dry, and 0.10 g of fresh nodule tissue was used for the assay. Nitrogenase activity was expressed as the ethylene production rate and converted to an ethylene production rate per unit nodule fresh weight.

### 2.9. Reverse-Transcription Quantitative PCR (RT-qPCR) Validation

To verify the reliability of RNA sequencing (RNA-seq) results, representative DEGs from both the host and rhizobial sides were selected for RT-qPCR assays (host candidates could include *ENOD93*, leghemoglobin, SWEET transporters, and representative amino-acid/peptide transport genes; rhizobial candidates could include *nifH*/*nifD*, cbb3-type oxidase-related genes, and key transport/chemotaxis genes). Primer sequences are listed in Supplementary Table S3. Total RNA was reverse transcribed into cDNA using PrimeScript™ RT reagent Kit with gDNA Eraser (Perfect Real Time) (Code No. RR047A; Takara Bio, Kusatsu, Shiga, Japan). Host-side expression was normalized to the reference gene *MtACTIN*, and rhizobial-side expression was normalized to *rpoD*. Each treatment included three biological replicates, and each sample was measured with three technical replicates. Relative expression was calculated using the 2^−ΔΔCt^ method [29], and results are presented as mean ± SEM of the three biological replicates.

## 3. Results

### 3.1. Phenotypic Differences in Alfalfa Induced by Inoculation with Two Rhizobial Strains

**Figure 1:**
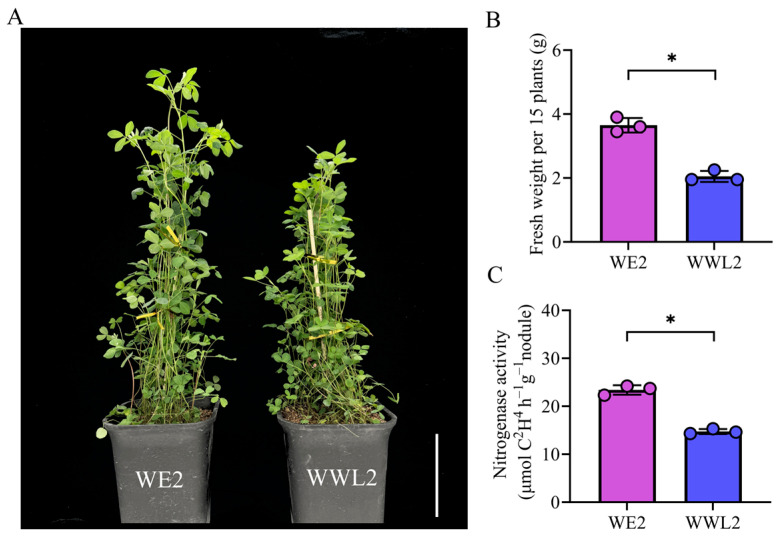
Phenotypic differences in 21 dpi nodules and plants (*WE2* vs. *WWL2*). (**A**) Representative plant phenotypes at 21 days post-inoculation (dpi); scale bar, 10 cm. (**B**) Shoot fresh weight (g) per biological replicate (each replicate is a pooled sample of 15 plants); *n* = 3 independent biological replicates. (**C**) Nitrogenase activity measured by the acetylene reduction assay (ARA) using pooled nodules from each biological replicate; *n* = 3 independent biological replicates. Data are shown as mean ± SEM; dots indicate biological replicates (*n* = 3). Statistical significance between *WE2* and *WWL2* was assessed using a two-tailed unpaired Welch’s *t*-test. * indicates *p* < 0.05.

When nodules were collected at 21 dpi, we observed significant differences in plant phenotypes induced by the two *S. meliloti* strains (*WE2* and *WWL2*) (Figure 1A). Specifically, both shoot fresh weight (0.243 ± 0.015) and nitrogenase activity (23.4 ± 1.0) were significantly higher in *WE2* than in *WWL2* (Figure 1B,C). Based on this clear phenotypic divergence, we performed host–symbiont dual transcriptome sequencing on nodule tissues harvested at the same time point to characterize transcriptional differences associated with the phenotypic differences.

### 3.2. Overall Distribution of Transcriptome Data and Sample Consistency (PCA)

**Figure 2:**
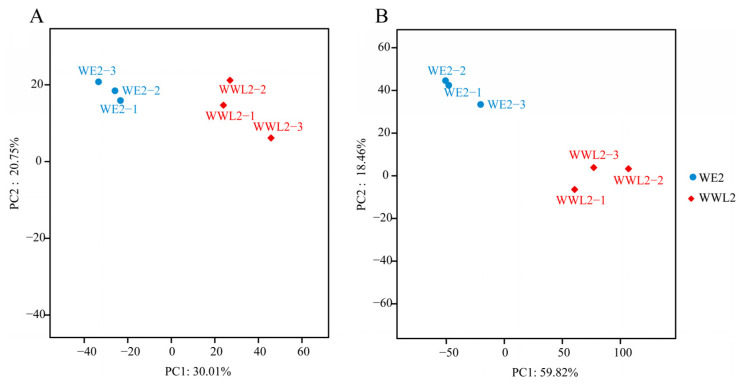
Quality control of dual transcriptome data. (**A**) PCA of alfalfa-side samples. (**B**) PCA of rhizobial-side samples. Percent variance explained by PC1 and PC2 is shown on the axes.

Principal component analysis showed tight clustering of biological replicates and a clear separation between *WE2* and *WWL2* on both the host and rhizobial transcriptomes. We performed PCA separately for the alfalfa and rhizobial expression profiles (Figure 2). For the alfalfa side, the three biological replicates within the same inoculation treatment (*WE2* or *WWL2*) clustered tightly, whereas samples from the two treatments were clearly separated in principal component space (Figure 2A); PC1 and PC2 explained 30.01% and 20.75% of the total variance, respectively. For the rhizobial side, PCA similarly showed good replicate consistency and clear separation between treatments (Figure 2B), with PC1 and PC2 explaining 59.82% and 18.46% of the total variance, respectively. These results indicate that inoculation with different rhizobial strains caused pronounced shifts in overall transcriptional profiles at both the host and symbiont levels, providing a robust basis for subsequent differential expression and functional enrichment analyses.

### 3.3. Differential Expression and Functional Enrichment in the Host (Alfalfa) (WE2 vs. WWL2)

#### 3.3.1. Overview of Host Differentially Expressed Genes (Volcano Plot/Number of DEGs)

**Figure 3:**
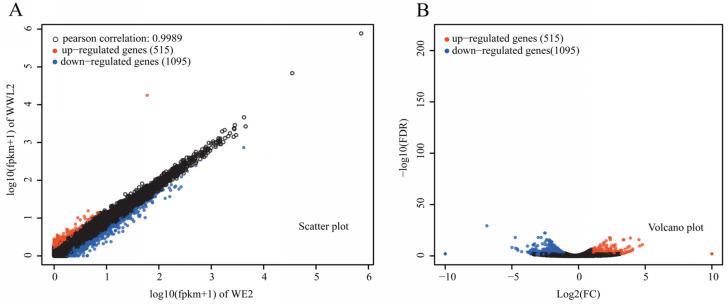
Overview of differential expression on the alfalfa side. (**A**) Scatter plot of expression levels for *WE2* and *WWL2* (log_10_(FPKM + 1)); (**B**) volcano plot of DEGs. In these plots, black circles represent genes with no significant differential expression. DEGs were defined using FDR < 0.05 and |log_2_FC| ≥ 1; log_2_FC was calculated as log_2_(*WWL2*/*WE2*).

Host DEGs were skewed toward higher expression in *WE2* than in *WWL2* at 21 dpi (Figure 3). Using FDR < 0.05 and |log_2_FC| ≥ 1, we identified 1610 host DEGs between *WE2* and *WWL2*, with 1095 higher in *WE2* and 515 higher in *WWL2* (Figure 3; Supplementary Table S4). This asymmetrical gene upregulation suggests that, compared with *WWL2*, *WE2* inoculation sustains a broader host transcriptional program during the mature nodule stage, particularly for genes involved in transmembrane transport, redox and heme-binding functions, and nodule development and microaerobic homeostasis. Compared with *WWL2*, *WE2* inoculation was associated with higher expression of many host genes involved in transmembrane transport, redox/heme-binding functions, and microaerobic homeostasis during the mature nodule stage.

#### 3.3.2. GO Enrichment: Transport, Redox, and Nodule-Development-Related Processes

To clarify the functional tendencies of DEGs, we performed GO classification and enrichment analyses for alfalfa-side DEGs (Supplementary Figures S1–S4). GO results showed significant enrichment of DEGs in terms related to transmembrane transport/substance transport, redox processes, heme/oxygen-related binding and metabolism, and energy metabolism. Notably, within these GO categories closely associated with mature nodule function, downregulated genes accounted for a higher proportion (Supplementary Figure S5), suggesting that these processes are overall more strongly expressed in mature nodules induced by *WE2* and are relatively weakened under *WWL2*. Overall, GO enrichment indicates that host-side differences are tightly linked to resource supply, metabolic adjustment, and oxygen environment-related regulation.

**Figure 4:**
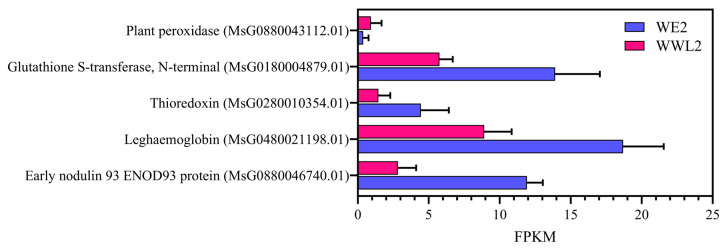
Host module for nodule function and microaerobic/redox homeostasis. Nodules were harvested at 21 days post-inoculation from alfalfa plants inoculated with *Sinorhizobium meliloti* strains *WE2* or *WWL2* (*n* = 3 biological replicates per treatment). This figure summarizes representative alfalfa DEGs associated with mature nodule function and microaerobic/redox homeostasis (including ENOD/leghemoglobin and antioxidant/redox-related genes). For each gene, expression level (FPKM) and differential-expression statistics (log_2_FC and FDR) are shown; log_2_FC is calculated as log_2_(*WWL2*/*WE2*) (log_2_FC > 0 indicates higher expression in *WWL2* relative to *WE2*). Differential expression was calculated from raw counts using edgeR (see Methods).

**Figure 5:**
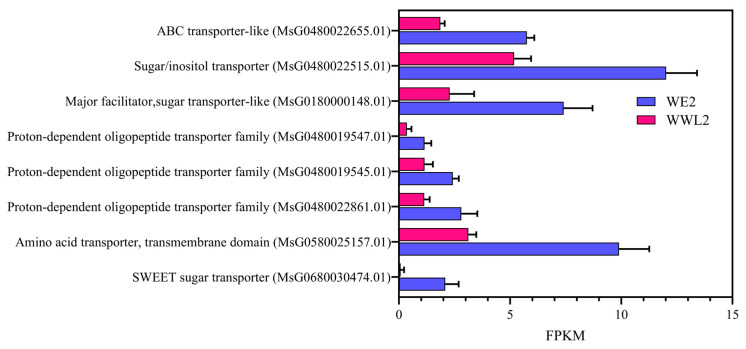
This figure shows 21 dpi alfalfa nodules induced by *WE2* and *WWL2* (*n* = 3). Representative host DEGs related to sugar transport and amino acid/peptide transport are shown with FPKM and differential-expression metrics (log_2_FC, FDR); log_2_FC = log_2_(*WWL2*/*WE2*). DE analysis was performed with edgeR (see Methods).

At the gene level, representative DEGs showed expression shifts that matched the enriched GO themes, linking functional enrichment to specific host nodes (Figure 4 and Figure 5; Supplementary Figures S6 and S7; Table 1). For example, the mature nodule marker genes *ENOD93* and leghemoglobin, together with transport genes including SWEET and representative amino acid or peptide transport genes, were higher in *WE2* than in *WWL2* (Figure 4 and Figure 5; Supplementary Figures S6 and S7; Table 1).

#### 3.3.3. KEGG Enrichment: Differential Responses in Metabolic and Signaling Pathways

We further performed KEGG pathway enrichment analysis for DEGs (Supplementary Figures S8–S11). Overall, enriched pathways included not only those related to metabolism and energy conversion but also plant signal transduction pathways; among them, hormone signaling, MAPK signaling, and α-linolenic acid metabolism were prominently enriched (Supplementary Figure S8).

At the KEGG category-statistics level, both up- and downregulated DEGs were distributed across multiple metabolism-related categories, including entries associated with sugar/carbon metabolism, nitrogen metabolism, and energy metabolism (e.g., glycolysis/gluconeogenesis, pyruvate metabolism, oxidative phosphorylation, nitrogen metabolism) (Supplementary Figure S9). Bubble plots for direction-specific subsets further refined these signaling and metabolic pathways: Supplementary Figure S10 includes entries such as pentose phosphate pathway, arginine biosynthesis, and glycolysis/gluconeogenesis, whereas Supplementary Figure S11 includes oxidative phosphorylation, pyruvate metabolism, and biosynthesis of amino acids. Collectively, enriched pathways were mainly distributed across carbon metabolism, nitrogen/amino-acid metabolism, and energy metabolism categories (Supplementary Figures S9–S11).

In addition, the pathway–gene network illustrates connections between pathway nodes and the DEGs they contain (Supplementary Figure S12), providing a basis for subsequent identification of key node genes and integration with representative gene modules.

#### 3.3.4. Representative DEGs: Nodes Related to Microaerobic Homeostasis and Substrate Exchange

Hierarchical clustering of alfalfa DEGs based on standardized expression produced a heat map with two major expression clusters (Supplementary Figure S13). One cluster exhibited higher expression in *WE2* and lower expression in *WWL2* across biological replicates, whereas the other cluster showed higher expression in *WWL2* and lower expression in *WE2*. Genes annotated to mature nodule function, microaerobic homeostasis, and substrate exchange were predominantly found in the cluster with higher expression in *WE2* (Supplementary Figure S13; Table 1).

#### 3.3.5. Enhanced Host Modules Related to “Substrate Supply-Microaerobic Homeostasis”

To focus on host DEGs directly linked to symbiotic functional output during the mature nodule stage, we organized alfalfa DEGs into four functional modules: nodule function and microaerobic homeostasis; substrate exchange and transmembrane transport; hormone signaling and transcriptional regulation; and immunity/recognition. For each module, classic marker genes and representative genes with large differential changes are summarized in Figure 4 and Figure 5 and Supplementary Figures S6 and S7, together with expression levels (FPKM) and differential metrics (log_2_FC, FDR; log_2_FC > 0 indicates relative upregulation in *WWL2*).

As shown in Figure 4 and Figure 5 and Supplementary Figures S6 and S7 (together with quantitative summaries of key genes in Table 1), compared with *WWL2*, *WE2* nodules exhibited higher expression of the mature nodule function/microaerobic homeostasis markers *ENOD93* and leghemoglobin, along with a consistent enhancement of multiple antioxidant and redox-regulation-related genes. Within the substrate-exchange module, SWEET-family sugar transporters and representative amino-acid/peptide transport-related genes were overall more highly expressed in *WE2*. Together, these modules show higher expression of mature nodule markers (e.g., *ENOD93* and leghemoglobin) and multiple transport/redox-related genes in *WE2* than in *WWL2* at 21 dpi. In addition, Supplementary Figures S6 and S7 summarize representative genes related to hormone/transcriptional regulation and immune recognition (e.g., auxin-related factors, receptor-like proteins, and defense regulators), providing supplementary context for differences in “symbiosis maintenance-homeostasis regulation” during maturity. We next examined corresponding expression differences on the rhizobial side (Section 3.4).

Beyond *ENOD93* and leghemoglobin, multiple substrate-input and transmembrane transport nodes were synchronously enhanced under *WE2*, including SWEET (*MsG0680030474.01*, log_2_FC = −4.60, FDR = 1.30 × 10^−7^), an MFS sugar transporter (*MsG0180000148.01*, log_2_FC = −1.69, FDR = 1.09 × 10^−6^), and a Sugar/Inositol transporter (*MsG0180005632.01*, log_2_FC = −1.03, FDR = 2.51 × 10^−3^) (Supplementary Table S5).

Meanwhile, redox-homeostasis-related genes were also consistently enhanced, such as quinone oxidoreductase (*MsG0680030763.01*, log_2_FC = −2.51, FDR = 1.13 × 10^−11^), thioredoxin (*MsG0280010354.01*, log_2_FC = −1.61, FDR = 6.22 × 10^−5^), and GST (*MsG0280009765.01*, log_2_FC = −1.28, FDR = 7.55 × 10^−4^). Together, these results strengthen the evidence chain for maintenance of microaerobic homeostasis in mature nodules from the perspective of “transport-redox regulation” (Supplementary Table S5). A more complete list of related DEGs is provided in Supplementary Table S6.

On the host side, DEGs associated with substrate exchange and maintenance of redox homeostasis showed a consistent enhancement under *WE2* inoculation. In addition to the core anchors for mature nodule/microaerobic homeostasis (*ENOD93* and leghemoglobin) listed in Table 1, we further summarized quantitative expression of “extended supporting nodes” related to sugar input, amino-acid/peptide transmembrane transport, and antioxidant/redox processes in Supplementary Table S5. The results show that multiple sugar-transport genes (e.g., an MFS sugar transporter and a Sugar/Inositol transporter), amino-acid/peptide transport genes, and redox-homeostasis-related genes were overall more highly expressed under *WE2* (Supplementary Table S2), consistent with the module directions shown in Figure 4 and Figure 5; Supplementary Figures S8 and S9. A more complete list of relevant DEGs is provided in Supplementary Table S6.

### 3.4. Differential Expression and Functional Modules on the Symbiont (Rhizobium) Side (WE2 vs. WWL2)

#### 3.4.1. Overview of Rhizobial DEGs (Number and Direction)

**Figure 6:**
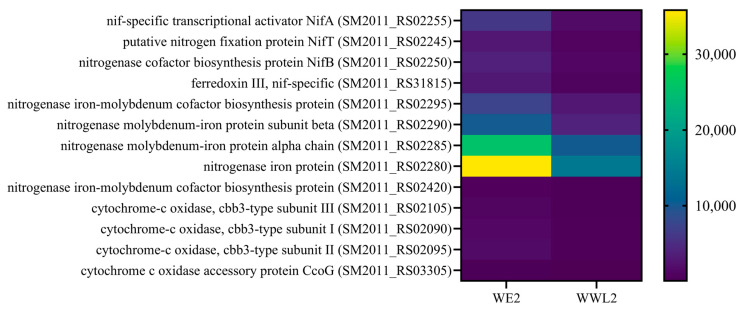
Nitrogen fixation and microaerobic respiration. Nodules were harvested at 21 dpi from plants inoculated with *WE2* or *WWL2* (*n* = 3). Representative rhizobial genes (*nif*/*fdx* and *cbb3*-related respiration genes) are shown with RPKM, log_2_FC, and FDR; log_2_FC = log_2_(*WWL2*/*WE2*).

To facilitate interpretation of functional allocation differences at the mature nodule stage from the symbiont perspective, we grouped rhizobial DEGs into four functional modules based on KEGG, GO, and COG annotations: (1) nitrogen fixation and microaerobic respiration; (2) nodulation signals and surface structures; (3) chemotaxis and motility; and (4) transport and nutrient acquisition. For each module, marker genes and representative DEGs with large changes are summarized in Figure 6 and Supplementary Figures S14–S16 (log_2_FC > 0 indicates higher expression in *WWL2*). Complete module-specific DEG lists are provided in Supplementary Table S7A–D, and Table S7B summarizes genes related to microaerobic respiration and electron transfer.

As shown in Figure 6 and Supplementary Figures S14–S16, genes related to nitrogen fixation (e.g., *nifA*, *nifH*, *nifD*) and microaerobic respiration or electron transfer (e.g., *fix* genes and cbb3-type terminal oxidase related genes) showed higher expression in *WE2* than in *WWL2*. In contrast, *WWL2* showed more upregulated genes in modules related to nodulation signals and surface structures (e.g., *nod*/*noe* and *exo*/*syr*), chemotaxis and motility (e.g., *che*/*fli*/*flg*/*mot*), and transport and nutrient acquisition (e.g., multiple ABC/MFS transporters and nutrient uptake-related genes).

Rhizobial differential expression was also analyzed, with *WWL2* vs. *WE2* as the comparison. DEGs were predominantly upregulated (1787 upregulated and 379 downregulated; Supplementary Figure S17), indicating that the two strains were operating under markedly different physiological programs within nodules. Given that sampling was performed at 21 dpi mature nodules, enrichment of upregulated terms in motility/environment sensing or nodulation-signal modules often suggests that bacteria may not have fully entered a stable bacteroid-like nitrogen-fixing state; therefore, functional annotation is required to further interpret differences in their “stage-specific programs.”

#### 3.4.2. Nitrogen Fixation and Microaerobic Respiration Genes Are Overall Higher in *WE2*

GO classification indicated that rhizobial DEGs mainly involve metabolic processes, transmembrane transport, redox functions, and responses to stimuli (Supplementary Figure S18). Further GO enrichment suggested that, in the context of mature nodules, terms related to nitrogen fixation/electron transfer/microaerobic respiration were more prominent under *WE2*, whereas terms associated with behavioral regulation, chemotaxis/motility, and surface structures were more pronounced under *WWL2* (Supplementary Figures S19–S21).

Closer inspection showed that transmembrane transport (especially ABC transport systems) and ion/proton transport terms were broadly upregulated in *WWL2*, suggesting that *WWL2* places greater emphasis on nutrient acquisition and environmental adaptation within the nodule microenvironment. Meanwhile, multiple motility/chemotaxis-related genes were overall upregulated in *WWL2*, which contrasts with the common pattern that mature bacteroids suppress flagellar/chemotaxis systems, which contrasts with reports that motility/chemotaxis genes are typically downregulated in mature bacteroids. Here, *WWL2* showed higher expression of multiple motility/chemotaxis genes at 21 dpi (e.g., *cheR*: log_2_FC = 1.8550, FDR = 0.004203; *fliG*: log_2_FC = 2.7403, FDR = 8.71 × 10^−6^; *fliI*: log_2_FC = 2.5057, FDR = 2.98 × 10^−6^).

#### 3.4.3. *WWL2* Shows More Prominent Nodulation Signal/Surface-Structure Genes

KEGG enrichment further revealed differences in functional emphasis between the two strains within mature nodules (Supplementary Figure S22). In the *WE2* inoculation group, pathways/gene modules directly related to nitrogen fixation (the *nif* gene cluster and Fix-associated electron transfer and microaerobic respiration modules) were more enriched and showed overall stronger expression. In contrast, the *WWL2* group showed more prominent, predominantly upregulated pathways related to environmental information processing (e.g., two-component systems, ABC transport systems, and chemotaxis/motility modules). In addition, several nodulation-signal-related genes were significantly upregulated in *WWL2*, including *nodI* (log_2_FC = 2.6863, FDR = 1.51 × 10^−4^), *nodB* (log_2_FC = 2.6139, FDR = 2.69 × 10^−3^), and *nodN* (log_2_FC = 13.3038, FDR = 6.51 × 10^−3^), suggesting that *WWL2* may maintain relatively high expression of genes involved in nodulation signaling surface polysaccharide regulation during maturity (e.g., *exoZ*: log_2_FC = 3.9689, FDR = 3.94 × 10^−7^). Direction-specific subset enrichment results are shown in Supplementary Figures S23 and S24.

The KEGG pathway–gene network illustrates connections among energy metabolism, transport, and regulatory pathways (Supplementary Figure S25). Some node genes connect both nitrogen fixation/respiration and substrate uptake modules, suggesting that rhizobial transcriptional emphasis and adaptation strategies differ between treatments.

#### 3.4.4. *WWL2* Shows More Prominent Chemotaxis/Motility and Environmental-Response Regulation

The heat map shows that most representative DEGs have higher standardized expression in *WE2*, including many genes annotated to nitrogen fixation and microaerobic respiration (*nif*/*fix* and *cbb3*-type terminal oxidase-related genes). In contrast to this main cluster, the top two genes in the heat map display an opposite pattern and both encode membrane permease components of substrate-uptake transmembrane transport systems: the TRAP transporter large permease subunit *SM2011_RS08125* (TRAP transporter large permease, log_2_FC = +14.758, FDR = 1.24 × 10^−13^) and the ABC transporter permease *SM2011_RS29460* (ABC transporter permease, log_2_FC = +5.218, FDR = 7.76 × 10^−16^) are markedly higher in *WWL2*; their associated components (e.g., the TRAP substrate-binding protein *RS08120* and the ABC ATPases *RS29465*/*RS29470*) are synchronously upregulated as well, suggesting that *WWL2* preferentially activates a “substrate uptake/transport” strategy within nodules, whereas *WE2* preferentially shows higher expression of symbiotic output modules such as nitrogen fixation–microaerobic respiration (Figure 6). Overall, this heat map visualizes the transcriptional strategy divergence between the two strains in mature nodules: *WE2* is biased toward symbiotic output modules (nitrogen fixation–microaerobic respiration), whereas *WWL2* is biased toward substrate uptake and adaptive transport nodes, summarizing strain-associated expression differences in mature nodules (Supplementary Figure S26).

### 3.5. Cross-Partner Integration: Coordinated Changes Between Host Supply/Microaerobic Homeostasis and Rhizobial Expression of Nitrogen Fixation Genes

**Figure 7:**
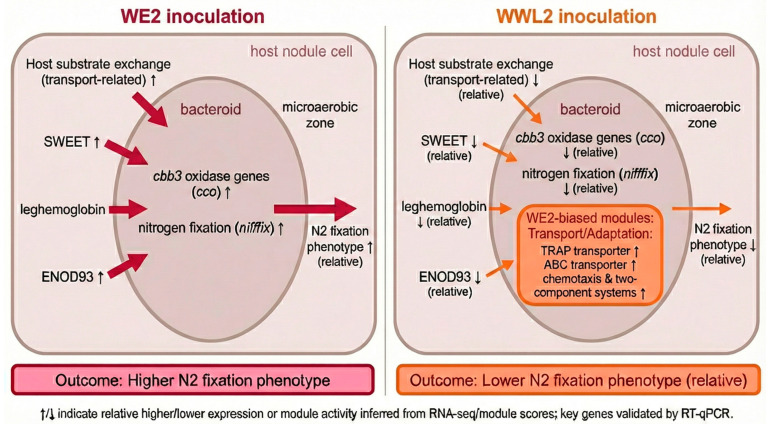
Working framework proposed based on integrated dual transcriptomes (schematic). The framework highlights coordinated changes between host nodes related to substrate exchange and microaerobic homeostasis (*ENOD93*, leghemoglobin, SWEET, and amino-acid/peptide transport) and rhizobial nitrogen fixation/microaerobic respiration modules within the 21 dpi mature nodule window. This framework is used to explain phenotypic differences observed in this study (Figure 1); functional indicators such as nitrogenase activity (ARA) can be used to test framework predictions (Section 3.1; Figure 1C). ↑/↓ indicate up-/downregulation of the relevant gene sets in the comparison of *WE2* vs. *WWL2* (or vice versa) (21 dpi).

We integrated key nodes from both partners to build a working model (Figure 7). Under *WE2*, host mature nodule markers and substrate supply-related nodes showed higher expression, and rhizobial genes for nitrogen fixation and microaerobic respiration were more pronounced. Under *WWL2*, multiple host exchange-related nodes were lower, whereas rhizobial processes related to transport, chemotaxis and motility, and environmental information processing were more enriched (Figure 7).

#### 3.5.1. Module Scores and Key Gene Evidence Support Coordinated Cross-Partner Enhancement

We calculated module scores as the mean of per-gene z scored expression values within each predefined gene set to summarize relative module level expression patterns across samples (Table 2). These scores are descriptive summaries based on per-gene z scores and are not interpreted as inferential statistics or absolute expression units. The host “substrate supply/microaerobic homeostasis” module score was overall higher under *WE2* (0.930 ± 0.247) and lower under *WWL2* (−0.930 ± 0.302). On the rhizobial side, the “nitrogen fixation/microaerobic respiration” module showed a parallel shift (*WE2*: 0.938 ± 0.117; *WWL2*: −0.938 ± 0.241). Conversely, *WWL2* scored higher for “nodulation signals and surface structures” (0.864 ± 0.294 vs. −0.864 ± 0.170), “chemotaxis and motility” (0.886 ± 0.312 vs. −0.886 ± 0.530), and “transport and nutrient acquisition” (0.841 ± 0.318 vs. −0.841 ± 0.211) modules (Table 2). Moreover, the host “substrate supply/microaerobic homeostasis” module score was positively correlated with the rhizobial “nitrogen fixation/microaerobic respiration” module score across all samples (Pearson r = 0.923, *p* = 0.00868, *n* = 6).

#### 3.5.2. RT-qPCR Validation of Key Gene-Expression Trends

**Figure 8:**
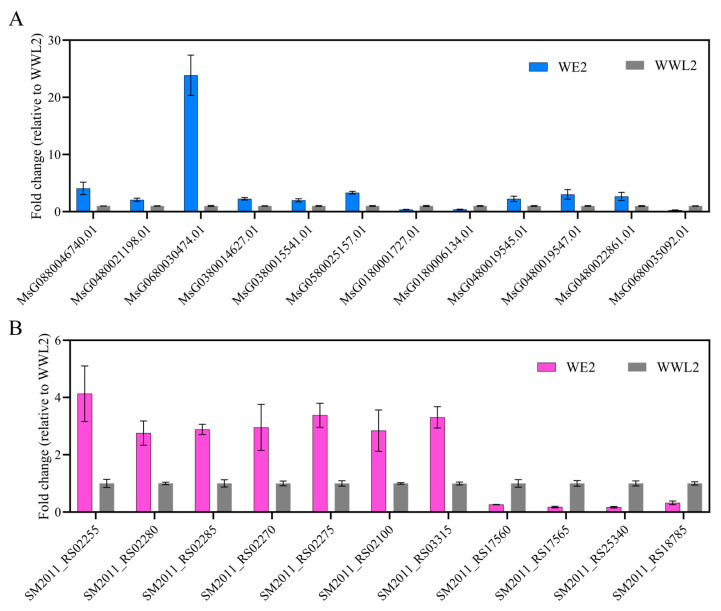
RT qPCR validation of representative host and rhizobial genes. Nodules were harvested at 21 dpi from plants inoculated with strains *WE2* or *WWL2*. (**A**) Host gene expression was normalized to MtACTIN. (**B**) Rhizobial gene expression was normalized to rpoD. Relative expression was calculated using the 2^−ΔΔCt^ method. Data are presented as mean ± SEM from three biological replicates (*n* = 3). Each biological replicate was measured with three technical replicates.

RT-qPCR results showed that key nodes on the host side—*ENOD93* (*MsG0880046740.01*), leghemoglobin (*MsG0480021198.01*), SWEET (*MsG0680030474.01*), and representative transport genes—and key nodes on the rhizobial side such as *nifH* (*SM2011_RS02280*)/*nifD* (*SM2011_RS02285*) displayed trends consistent with RNA-seq (Figure 8; mean ± SEM, *n* = 3). These expression differences in key nodes match the direction of between-treatment differences in nitrogen fixation efficiency and growth phenotypes, consistent with an association between “key coupled nodes” and “functional output” in the proposed framework (Figure 1, Figure 4 and Figure 5; Supplementary Figures S6 and S7).

On the host side, nodes related to substrate exchange and microaerobic homeostasis in mature nodules showed a consistent enhancement under *WE2*: mature nodule/microaerobic maintenance markers such as *ENOD93* and leghemoglobin were higher in *WE2* in both qPCR and RNA-seq, and SWEET and amino-acid/peptide transport-related genes were overall more active (Table 1; Figure 4 and Figure 5; Supplementary Figures S4, S6 and S7). On the rhizobial side, the nitrogen fixation and microaerobic respiration module directly linked to symbiotic output showed the same directional change, with core *nif*/*fix* genes and *cbb3*-type terminal oxidase-related genes overall higher in *WE2*, whereas *WWL2* more prominently featured genes related to chemotaxis/motility, two-component systems, and multiple transport and nutrient-acquisition processes (Figure 6; Supplementary Figures S14–S16 and S22–S26). This concordant pattern—enhanced host substrate supply and oxygen buffering capacity alongside increased rhizobial expression of nitrogen fixation and microaerobic respiration genes—supports coordinated functional emphasis between the host and the symbiont across key functional modules during the mature nodule stage.

## 4. Discussion

Starting from the phenotypic divergence between *WWL2*- and *WE2*-inoculated plants (Figure 1), this study compared host–symbiont dual transcriptomes within the 21 dpi mature nodule window and built an evidence chain around “phenotypic differences → bilateral transcriptional responses → mechanistic interpretation” [30,31]. Overall, host-side differences were concentrated in substrate supply/transport and maintenance of microaerobic homeostasis, whereas rhizobial-side differences were concentrated in resource allocation between the microaerobic respiration and nitrogen fixation program and environmental adaptation programs [32,33,34]. Their coupling forms a mechanistic main line from “host supply-microaerobic homeostasis” to “rhizobial expression of nitrogen fixation genes,” providing a coherent explanation consistent with phenotype direction. Below, we discuss key nodes within this coupled framework, including hormone-immunity rebalancing, regulation of the redox microenvironment, and regulation of the rhizobial nitrogen fixation program [33,35,36,37,38].

### 4.1. Host Hormone- and Defense-Related Differences and Their Relationship to Mature Nodule Maintenance

At the mature nodule stage (21 dpi), the primary physiological tasks focus on maintaining a microaerobic environment and facilitating carbon–nitrogen exchange. It is also critical to prevent excessive immune responses that might otherwise disrupt the symbiosis [32,33,35]. Our results show that alfalfa DEGs are significantly enriched in plant hormone signaling and MAPK pathways. This enrichment suggests that different rhizobial strains may alter the functional homeostasis of nodules through these complex signaling networks. Notably, multiple auxin-responsive (SAUR) genes were downregulated under *WWL2*, implying weakened growth regulation associated with nodule tissue maintenance/sink function. In addition, a small number of immune-related receptor-like genes (TIR/NB-ARC class) showed directional changes, suggesting that hosts may adopt different immune “threshold settings” under different strains, thereby influencing symbiont differentiation and the nitrogen fixation program [33,35,36].

### 4.2. Differences Related to Redox Homeostasis and Secondary Metabolism: Implications for Regulation of the Nodule Microenvironment

The enrichment of redox-related pathways and secondary metabolism in the host suggests differences in redox/ROS-associated processes between treatments. These pathways may contribute to regulation of the nodule microenvironment and are discussed here as hypotheses for future validation [38].

### 4.3. Differences in Symbiont Infection/Adaptation-Related Expression and Activation of the Nitrogen Fixation Program

In mature nodules, bacteroids typically suppress motility/chemotaxis systems and strengthen microaerobic respiration and nitrogenase expression [34,39]. We observed a clear divergence in rhizobial gene expression between the two treatments. *WE2* aligns more closely with the nitrogen fixation program of mature bacteroids, characterized by elevated expression of core modules such as *nif*, *fix*, and *cbb3*. Simultaneously, systems associated with free-living states, including motility and chemotaxis, are relatively suppressed in the context of nodule residence. In contrast, *WWL2* showed more active expression of genes related to chemotaxis/flagella, ABC transporters, and environmental information processing. These results suggest that differences between the two strains are more likely reflected in differentiation and energy-use efficiency after entering nodules, thereby affecting establishment and maintenance of the mature nitrogen fixation program [33,34].

### 4.4. Summary of Transcriptional Evidence for Phenotypic Differences and Study Limitations

At 21 dpi, we measured nodule and plant phenotypes (Figure 1), including fresh weight above ground and nitrogen fixation functional indicators (ARA). Phenotypic results indicate differences in nodule maturity and nitrogen fixation output induced by the two strains (see Section 3.1). Based on these observations, dual transcriptome data provide a cross-species, coherent molecular explanation: under *WE2*, host-side key nodes such as *ENOD93*, leghemoglobin, SWEET, and amino-acid/peptide transporters were higher, indicating stronger substrate exchange and maintenance of microaerobic homeostasis at the mature nodule stage [28,32,33,34]. Meanwhile, rhizobial microaerobic respiration–nitrogen fixation modules (*nif*/*fix*/*cbb3*, etc.) were more prominent, whereas under *WWL2* the rhizobium was biased toward processes related to transport/chemotaxis and environmental adaptation. Overall, at 21 dpi, *WE2* nodules showed higher nitrogenase activity (ARA) and higher expression of rhizobial *nif*/*fix*/*cbb3* genes and host microaerobic-homeostasis/transport genes than *WWL2*, indicating a stronger nitrogen fixation-associated transcriptional signature at this stage [27]. Although module scores are derived from predefined gene sets and serve as descriptive summaries of coordinated expression rather than independent quantitative measurements, we performed a pragmatic check using independent readouts collected from the same biological replicates. The direction of between treatment differences in key module scores agreed with phenotypic indicators at 21 dpi, including higher nitrogenase activity measured by the acetylene reduction assay and higher shoot fresh weight under *WE2* (Figure 1). The same direction was also supported by RT qPCR measurements of representative marker genes within these modules (Figure 7). Nevertheless, module scoring was not evaluated on an external cohort or additional time points, and further validation in independent datasets will be required to assess generalizability.

It should be noted that differences in nodule maturity/bacteroid proportion observed under *WWL2* can themselves be viewed as part of the interaction-state differences after strain inoculation. With a design comparing only a single time point (21 dpi), this study interprets the observed differences as integrated output-state differences between two interaction systems at the same sampling time; accordingly, caution is warranted against attributing all differences to a single regulatory step. Because samples were collected at a single time point, we cannot exclude the possibility that some expression differences reflect asynchronous nodule development or bacteroid differentiation rates between strains. Therefore, our interpretations are limited to transcriptional associations at the sampled stage.

In addition, host transcripts dominate dual transcriptomes from nodule tissues, resulting in a relatively low proportion of bacterial reads in total reads (1.64–5.04% in this study; Supplementary Table S1). This phenomenon mainly reflects sample composition rather than sequencing quality, but it also implies that detection sensitivity for low-abundance bacterial transcripts may be limited. Therefore, symbiont-side mechanistic inferences here focus on key modules and candidate genes that are directionally consistent, reproducible across replicates, and biologically interpretable, and are cross-validated against host-side signals.

As with many reference-based transcriptomic analyses, our symbiont side expression estimates may be influenced by the choice of mapping reference. In particular, rhizobial reads from *WE2* and *WWL2* were mapped to the *Sinorhizobium meliloti 1021* reference genome, which provides consistent gene identifiers and functional annotation across samples but may reduce sensitivity for strain unique genes or highly diverged loci. Therefore, our rhizobial side interpretations focus on conserved orthologous genes and modules with reproducible directional differences across biological replicates, and future strain specific genome assemblies and mapping will be needed to evaluate strain specific transcriptional programs.

### 4.5. Working Hypothesis Derived from Integrated Dual Transcriptomes: Differences in Functional Emphasis at the Mature Nodule Stage

Integrating results from both the host and the symbiont, we constructed a working framework (Figure 7) and used a tiered description to avoid overextending beyond the data: (Tier 1: data facts) compared with *WWL2*, *WE2* showed overall higher expression of host *ENOD93*/leghemoglobin and sugar and amino-acid transport-related genes, while rhizobial *nif*/*fix* and microaerobic respiration-related genes were more prominent; conversely, *WWL2* showed a weakening trend in mature nitrogen fixation related modules and a stronger bias toward environmental adaptation processes. (Tier 2: functional inference) these modules are typically involved in substrate supply, energy configuration, and maintenance of microaerobic homeostasis in mature nodules [32,33]. (Tier 3: phenotype correspondence) these molecular differences are consistent with the phenotypic differences we observed and can be further tested using functional indicators such as nitrogenase activity (ARA) (see Section 3.1; Figure 1C).

To evaluate this working framework beyond transcriptional associations, future studies can perform targeted functional tests on key nodes from both partners. Host candidates involved in substrate transport and oxygen buffering, together with symbiont *nif*, *fix*, and *cbb3* oxidase-related genes in *WE2* and *WWL2*, can be tested using genetic perturbation and complementation, followed by nitrogenase activity assays, nodule histology, and measurements of nodule oxygen status, redox state, and central carbon and nitrogen metabolites. These experiments would provide direct functional evidence and help assess the utility of candidate markers for inoculant screening.

### 4.6. Statistical Assumptions and Limitations

Our analyses were designed to identify reproducible differences between inoculation treatments and to prioritize candidates for downstream validation rather than to infer causality. Differential expression was evaluated with edgeR under a negative binomial framework using independent biological replicates, with TMM normalization, filtering of lowly expressed genes, and control of multiple testing using FDR adjusted *p* values together with an effect size cutoff (|log_2_FC| ≥ 1) [22,23].

Enrichment analyses tested over representation of differentially expressed genes in GO terms and KEGG pathways relative to the expressed background, with significance assessed using FDR-adjusted *p* values [24,25,40]. Phenotypic traits and nitrogenase activity were compared at the level of biological replicates (*n* = 3), which limits statistical power for modest effects and increases sensitivity to variability and outliers; therefore, the highlighted genes and pathways should be interpreted as associations that support a working model, and additional replicates, time points, and independent cohorts will be needed to assess robustness and generalizability.

## 5. Conclusions

Using two rhizobial strains with contrasting nitrogen fixation efficiency, we compared 21 dpi alfalfa nodules at both phenotypic and dual transcriptome levels. First, strain-dependent phenotypic differences were accompanied by consistent host transcriptional shifts related to substrate supply and transport and microaerobic homeostasis, including higher expression of *ENOD93*, leghemoglobin, and representative transport genes under *WE2*. Second, these host side patterns aligned with rhizobial expression programs, with higher expression of nitrogen fixation and microaerobic respiration genes in *WE2*, whereas *WWL2* showed relatively higher expression of genes related to transport, chemotaxis and motility, and environmental information processing. Third, integrating both partners, we propose a working framework and a prioritized set of candidate genes and pathways for downstream functional validation and inoculant screening. These conclusions are based on transcriptional associations at the sampled stage and should be tested by targeted perturbation and time course designs.

## Figures and Tables

**Table 1:** Summary of differential expression of key marker genes identified by dual transcriptome analysis (*WE2* vs. *WWL2*).

Category	GeneID	Name	Product	log_2_FC	FDR
Host (alfalfa)	*MsG0880046740.01*	*ENOD93*	Early nodulin 93 protein	−2.079	1.53 × 10^−10^
	*MsG0480021198.01*	leghemoglobin	Leghaemoglobin	−1.069	3.84 × 10^−3^
Rhizobium	*SM2011_RS02280*	*nifH*	nitrogenase iron protein	−1.341	2.19 × 10^−3^
	*SM2011_RS02275*	*fixA*	electron transfer flavoprotein subunit beta/FixA family protein	−1.539	1.47 × 10^−15^
	*SM2011_RS02090*	*ccoN*	cytochrome-c oxidase, cbb3-type subunit I	−1.815	4.63 × 10^−19^

**Table 2:** Module scores of host and rhizobial functional pathways in mature nodules under *WE2* vs. *WWL2* inoculation.

Module	*WE2* (Mean ± SD)	*WWL2* (Mean ± SD)
Host (alfalfa): substrate supply and microaerobic homeostasis	0.930 ± 0.247 a	−0.930 ± 0.302 b
Rhizobium: N fixation and microaerobic respiration	0.938 ± 0.117 a	−0.938 ± 0.241 b
Rhizobium: nodulation signal and surface structure	−0.864 ± 0.170 b	0.864 ± 0.294 a
Rhizobium: chemotaxis and motility	−0.886 ± 0.530 b	0.886 ± 0.312 a
Rhizobium: transport and nutrient acquisition	−0.841 ± 0.211 b	0.841 ± 0.318 a

Note: Different lowercase letters indicate significant differences between inoculation treatments (*WE2* vs. *WWL2*) (*p* < 0.05).

## Data Availability

The raw RNA-seq data generated in this study have been deposited in the NCBI Sequence Read Archive (SRA) under BioProject accession number PRJNA1406807.

## Supplementary Materials

The following supporting information can be downloaded at https://www.mdpi.com/article/10.3390/microorganisms14030571/s1, Figure S1: GO enrichment bubble plot for alfalfa DEGs (overall). The *x*-axis indicates the rich factor; bubble size represents the number of DEGs; color indicates the *p* value; Figure S2: GO enrichment bubble plot for alfalfa DEGs (directional subset 1), highlighting significant terms related to transcriptional regulation, hormone responses, and responses to biological stimuli; Figure S3: GO enrichment bubble plot for alfalfa DEGs (directional subset 2), enriched in terms related to transmembrane transport, ion transport, and electron transfer; Figure S4: GO functional classification of alfalfa DEGs. Numbers of up- and downregulated genes are shown across the three GO domains; Figure S5: Distribution of upregulated and downregulated alfalfa DEGs across BP/CC/MF categories (shown on a logarithmic scale); Figure S6: Hormones and transcriptional regulation (auxin and key signaling/transcription factors); Figure S7: Immune recognition-related genes (receptor-like proteins and defense regulation); Figure S8: KEGG enrichment bubble plot for alfalfa DEGs; Figure S9: KEGG pathway classification statistics for alfalfa DEGs (counts shown separately for upregulated and downregulated genes); Figure S10: KEGG enrichment bubble plot for alfalfa DEGs (directional subset 1); Figure S11: KEGG enrichment bubble plot for alfalfa DEGs (directional subset 2), including steroid biosynthesis, ubiquitin-mediated proteolysis, oxidative phosphorylation, and related pathways; Figure S12: KEGG pathway–gene association network for alfalfa DEGs. Squares represent pathways and circles represent genes; colors distinguish upregulated and downregulated genes; Figure S13: Clustering heat map of alfalfa DEGs; Figure S14: Nodulation signals and surface structures; Figure S15: Chemotaxis and motility; Figure S16: Transport and nutrient acquisition; Figure S17: Overview of differential expression on the rhizobial side; Figure S18: GO functional classification of rhizobial DEGs; Figure S19: GO enrichment bubble plot for rhizobial DEGs (overall), highlighting terms related to nitrogen fixation, electron transfer, and redox processes; Figure S20: GO enrichment bubble plot for rhizobial DEGs (transport/ion-transport related terms); Figure S21: GO enrichment bubble plot for rhizobial DEGs (behavior/stimulus-response related terms); Figure S22: KEGG enrichment bubble plot for rhizobial DEGs; Figure S23: KEGG enrichment bubble plot for rhizobial DEGs (subset 1); Figure S24: KEGG enrichment bubble plot for rhizobial DEGs (subset 2); Figure S25: KEGG pathway–gene association network for rhizobial DEGs. Squares represent pathways and circles represent genes; colors distinguish upregulated and downregulated genes. Figure S26: Clustering heat map of rhizobial DEGs; Table S1: Statistics of rhizobial read mapping (reference genome: *Sinorhizobium meliloti 1021*); Table S2: Gene sets for functional modules used in module score calculations. Table S3: Primers used in this study; Table S4: Summary of differentially expressed genes in 21 dpi nodules (*WWL2* vs. *WE2*); Table S5: Quantitative expression of extended supporting-node DEGs related to substrate exchange and microaerobic homeostasis on the alfalfa side (*WE2* vs. *WWL2*). Table S6: Differentially expressed genes associated with key functions in alfalfa nodules (excerpt); Table S7: Differentially expressed genes in key rhizobial functional modules (excerpt); Table S7A: DEGs related to nitrogen fixation (*nif*); Table S7B: DEGs related to microaerobic respiration/electron transfer (*FixA*/*FixB*/*FixH*/*FixQ* and cbb3 assembly); Table S7C: DEGs related to nodulation signaling (*nod*/*noe*/*nol*); Table S7D: DEGs related to chemotaxis/motility/flagella.

## Abbreviations

The following abbreviations are used in this manuscript:

ARA	Acetylene reduction assay
BP	Biological process (Gene Ontology)
CC	Cellular component (Gene Ontology)
cDNA	Complementary DNA
COG	Cluster of Orthologous Groups of proteins
DEGs	Differentially expressed genes
DNase	Deoxyribonuclease
dpi	Days post-inoculation
FC	Fold change
FDR	False discovery rate
FPKM	Fragments per kilobase of transcript per million mapped reads
GO	Gene Ontology
MAPK	Mitogen-activated protein kinase
MF	Molecular function (Gene Ontology)
MFS	Major facilitator superfamily
PCA	Principal component analysis
RefSeq	Reference Sequence database (NCBI)
RIN	RNA integrity number
RNA-seq	RNA sequencing
RPKM	Reads per kilobase of transcript per million mapped reads
RT-qPCR	Reverse-transcription quantitative PCR
SEM	Standard error of the mean
SRA	Sequence Read Archive
TMM	Trimmed mean of M-values
TRAP	Tripartite ATP-independent periplasmic transporter
UNG	Uracil-N-glycosylase
