# Supplementary material for: Dual-Transcriptome Dissection of the Mechanisms Underlying Alfalfa Phenotypic Differences Induced by Two Rhizobial Isolates
Source: Microorganisms. 2026 Mar 3;14(3):571. doi: 10.3390/microorganisms14030571 (PMC13029754; doi:10.3390/microorganisms14030571)
Supplement: Supplementary file 1 [file microorganisms-14-00571-s001.zip › microorganisms-4150422-supplementary.pdf]

Supplementary Materials

The Supplementary Materials include Supplementary Figures S1–S26 and Supplementary Tables S1–S7, providing additional information on data quality control, directional subset enrichment, an overview of alternative splicing events, and an extended list of candidate genes, among others. Key supplementary figures and tables (e.g., Table S7 and Table S1) are cited at the relevant locations in the Results/Discussion of the main text, while the remaining items are provided for reference as the complete dataset and detailed analytical results.

Table S1. Statistics of rhizobial read mapping (reference genome: Sm1021). Mapped rate (%) = Mapped reads / total clean reads per sample × 100.

Sample	Clean reads	Mapped reads	Mapped rate (%)
WE2-1	57,575,704	942,038	1.64
WE2-2	67,958,574	1,634,464	2.41
WE2-3	68,525,432	1,665,796	2.43
G9L8-1	71,128,818	3,350,146	4.71
G9L8-2	68,284,668	2,228,910	3.26
G9L8-3	68,586,238	3,454,356	5.04

Table S2. Gene sets for functional modules used in module score calculations.

Side	Module	GeneID	Gene/Abbrev	Brief functional description
Alfalfa	Microaerobic homeostasis / mature nodule marker	MsG0880046740.01	ENOD93	Mature nodule / maintenance of a microaerobic environment
	Microaerobic homeostasis / heme binding	MsG0480021198.01	leghemoglobin	Hemoglobin / buffering of microaerobic conditions
	Sugar transport / carbon input	MsG0680030474.01	SWEET	Sugar transporter
		MsG0180000148.01	MFS transporter	MFS sugar transporter
		MsG0180005632.01	Sugar/Inositol transporter	Sugar/inositol transport
	Amino acid/peptide transport	MsG0580025157.01	AA transporter	Amino acid transporter
		MsG0480022861.01	POT/PTR	Peptide transporter (POT/PTR)
		MsG0480019547.01	POT/PTR	Peptide transporter

				(POT/PTR)
		MsG0680030763.01	Quinone oxidoreductase	Quinone oxidoreductase (redox regulation)
	Redox / antioxidant	MsG0280010354.01	Thioredoxin	Thioredoxin (antioxidant defense)
		MsG0280009765.01	GST	Glutathione S-transferase (antioxidant defense)
Rhizobium	Nitrogen fixation and microaerobic respiration	SM2011_RS02255	nifA	Nitrogen fixation regulatory factor
		SM2011_RS02280	nifH	Nitrogenase Fe protein
		SM2011_RS02285	nifD	Nitrogenase MoFe subunit
		SM2011_RS31815	fdxB	nif-specific ferredoxin
		SM2011_RS02090	ccoN	cbb3-type cytochrome oxidase subunit I
		SM2011_RS02095	ccoO	cbb3-type cytochrome oxidase subunit II
		SM2011_RS02105	ccoP	cbb3-type cytochrome oxidase subunit III
		SM2011_RS23650	petA	bc1 complex Fe–S subunit
	Chemotaxis and motility	SM2011_RS17650	fliI	Flagellar export ATPase
		SM2011_RS17765	flgK	Flagellar hook-associated protein
		SM2011_RS18785	mcpU	Chemotaxis receptor protein
		SM2011_RS17570	cheR	Chemotaxis methyltransferase
		SM2011_RS17560	cheA	Chemotaxis histidine kinase
	Two-component	SM2011_RS16890	phoR	Phosphate-regulation

systems / regulation			sensor kinase
	SM2011_RS21775	ntrX	Nitrogen-regulation response regulator
	SM2011_RS21770	ntrY	Nitrogen-regulation sensor kinase
Transport and nutrient acquisition	SM2011_RS16900	pstC	Phosphate ABC transporter permease subunit
	SM2011_RS27170	ugpC	Glycerol-3-phosphate ABC ATPase
	SM2011_RS14015	urtE	Urea ABC transporter ATPase
	SM2011_RS25935	fhuF	Siderophore–iron reductase

Table S3. Primers used in this study.

Gene ID	Forward primer (5′–3′)	Reverse primer (5′–3′)	Product size (bp)
MsG0880046740.01	CAGCTTGAGAGAGAGAGAAG	TCCCAGAATCAGTGACTGAC	120
MsG0480021198.01	GAATGACTCCTATTGGCATG	GTGGATTATTGTGATGGGAG	120
MsG0680030474.01	TAAAGACCAAGAGTGGTGAC	GTTACCAGGACATAGATGTC	118
MsG0380015541.01	CTCTTGACCGGATCAAATCC	GAGAACTTTGAGAATCGCCG	120
MsG0580025157.01	TTGTACTTGGGATGACCAGC	ACGGCTTCTTCCATCTAGTG	120
MsG0180001727.01	GAGAAATTCTTGACACCCGC	CAAGAGAGCATCTCCAAAGG	119
MsG0180006134.01	CTCTAAGCACTACAGCAGTC	ATGTTGTCTTGAACCCACCC	109
MsG0480019545.01	GAACATCGGCTGAAATCAGG	ACTATCAGCGTCTGCCTATC	120
MsG0480019547.01	CAAGCAAGGGAGTTCTATGC	AATATCCGGTCGTAGATGGG	120
MsG0480022861.01	ACACGAAAGTAACGCGTCTG	AGCGATTGTCCAGTATCGTC	121
MsG0680035092.01	ACTTGGTGAACTATCTCGGG	AAAGGCTCCAAGCAATGGTG	110

Table S4. Summary of differentially expressed genes in 21-dpi nodules (G9L8 vs WE2).

Entity	No. of upregulated genes	No. of downregulated genes
Alfalfa（FPKM）	515	1095
Rhizobium（RPKM）	1787	379

Table S5. Quantitative expression of extended supporting-node DEGs related to substrate exchange and microaerobic homeostasis on the alfalfa side (*WE2* vs *WWL2*)

Module	GeneID	Functional description	*WE2*_mean	*WWL2*_mean	log_2FC (*WWL2*/*WE2*)	FDR	Direction
Sugar transport/carbon input	*MsG0680030474.01*	SWEET sugar transporter	2.086	0.085	-4.601	1.30×10^{-7}	Higher in *WE2*
Sugar transport/carbon input	*MsG0180000148.01*	MFS sugar transporter	7.413	2.291	-1.693	1.09×10^{-6}	Higher in *WE2*
Sugar transport/carbon input	*MsG0180005632.01*	Sugar/Inositol transporter	106.015	51.871	-1.031	2.51×10^{-3}	Higher in *WE2*
Amino acid/peptide transport	*MsG0580025157.01*	Amino acid transporter	9.906	3.127	-1.663	1.33×10^{-7}	Higher in *WE2*
Amino acid/peptide transport	*MsG0480022861.01*	Peptide transporter (POT/PTR)	2.817	1.143	-1.301	5.08×10^{-4}	Higher in *WE2*
Amino acid/peptide transport	*MsG0480019547.01*	Peptide transporter (POT/PTR)	1.159	0.363	-1.67	6.22×10^{-3}	Higher in *WE2*
Redox/antioxidation	*MsG0680030763.01*	Quinone oxidoreductase (redox regulation)	3.173	0.555	-2.513	1.13×10^{-11}	Higher in *WE2*
Redox/antioxidation	*MsG0280010354.01*	Thioredoxin (antioxidant)	4.439	1.451	-1.613	6.22×10^{-5}	Higher in *WE2*
Redox/antioxidation	*MsG0280009765.01*	Glutathione S-transferase (antioxidant)	11.3	4.665	-1.276	7.55×10^{-4}	Higher in *WE2*

Table S6. Differentially expressed genes associated with key functions in alfalfa nodules (excerpt).

Module	GeneID	log_2FC	Direction	FDR	Annotation (brief)
Microaerobic homeostasis (globin/leghemoglobin)	MsG0480021198.01	-1.069	down	3.84×10^{-3}	Leghaemoglobin(IPR001032)|Globin-like superfamily(IPR009050)|Globin/Protoglobin(IPR012292)
Hormone/auxin response（auxin/SAUR）	MsG0880042799.01	-1.828	down	4.39×10^{-6}	Small auxin-up RNA(IPR003676)
	MsG0480021349.01	-2.354	down	6.64×10^{-6}	Small auxin-up RNA(IPR003676)
	MsG0380016136.01	-10.414	down	7.51×10^{-5}	Small auxin-up RNA(IPR003676)
	MsG0880042793.01	-1.955	down	9.92×10^{-5}	Dormancy/auxin associated protein(IPR008406)
	MsG0480021346.01	-1.664	down	1.90×10^{-4}	Small auxin-up RNA(IPR003676)
	MsG0380017607.01	1.078	up	3.01×10^{-4}	Small auxin-up RNA(IPR003676)
	MsG0280006748.01	-1.159	down	3.27×10^{-4}	Dormancy/auxin associated protein(IPR008406)
	MsG0480021384.01	-10.019	down	7.55×10^{-4}	Small auxin-up RNA(IPR003676)
	MsG0280008548.01	-1.403	down	1.19×10^{-3}	Small auxin-up RNA(IPR003676)
	MsG0780035932.01	-3.088	down	1.76×10^{-3}	Small auxin-up RNA(IPR003676)
Redox/detoxification (peroxidase/GST, etc.)	MsG0180002005.01	1.740	up	1.96×10^{-6}	Glutamyl/glutaminyl-tRNA synthetase(IPR000924)|Aminoacyl-tRNA synthetase, class I, conserved site(IP

					R001412)丨Glutathione
	MsG0780040422.01	2.159	up	9.58 × 10⁻⁶	Glutaredoxin(IPR002109)丨Thioredoxin-like superfamily(IPR036249)
	MsG0380015507.01	9.937	up	4.61 × 10⁻⁵	Glutaredoxin(IPR002109)丨Thioredoxin-like superfamily(IPR036249)
	MsG0280010354.01	-1.613	down	6.22 × 10⁻⁵	Thioredoxin(IPR005746)丨Thioredoxin domain(IPR013766)丨Thioredoxin-like superfamily(IPR036249)
	MsG0180004879.01	-1.274	down	2.03 × 10⁻⁴	Glutathione S-transferase, N-terminal(IPR004045)丨Glutathione S-transferase, C-terminal(IPR004046)丨Glutathione S-transfer
	MsG0280009765.01	-1.276	down	7.55 × 10⁻⁴	Glutathione S-transferase, N-terminal(IPR004045)丨Thioredoxin-like superfamily(IPR036249)丨Glutathione S-transferase, C-te
	MsG0580026027.01	1.469	up	1.03 × 10⁻³	Thioredoxin(IPR005746)丨Thioredoxin domain(IPR013766)丨Thioredoxin, conserved site(IPR017937)丨Thioredoxin-like superfamily
	MsG0880043112.01	1.312	up	1.32 × 10⁻³	Plant peroxidase(IPR000823)丨Haem peroxidase, plant/fungal/bacterial(IPR002016)丨Haem peroxidase(IPR010255)
	MsG0180003792.01	1.034	up	1.99 × 10⁻³	Glutathione S-transferase, N-terminal(IPR004045)丨Thioredoxin-like superfamily(IPR036249)
	MsG0380017866.01	-1.229	down	1.99 × 10⁻³	Glutathione S-transferase, N-terminal(IPR004045)丨Prostaglandin E synthase 2, C-terminal(IPR034335)丨Thioredoxin-like supe
Immune/receptor regulation (TIR/NB-AR	MsG0080048967.01	-2.081	down	2.52 × 10⁻¹¹	WRKY domain(IPR003657)丨WRKY domain superfamily(IPR036576)

C/WRKY)					
	MsG0480023599.01	-10.861	down	5.71×10^{-8}	WRKY domain(IPR003657)丨WRKY domain superfamily(IPR036576)
	MsG0680032533.01	1.383	up	3.84×10^{-3}	Toll/interleukin-1 receptor homology (TIR) domain(IPR000157)丨NB-ARC(IPR002182)丨P-loop containing nucleoside triphosphate
	MsG0480023100.01	-1.739	down	4.39×10^{-6}	Toll/interleukin-1 receptor homology (TIR) domain(IPR000157)丨NB-ARC(IPR002182)丨P-loop containing nucleoside triphosphate
	MsG0580029679.01	-9.569	down	6.64×10^{-6}	Toll/interleukin-1 receptor homology (TIR) domain(IPR000157)丨Toll/interleukin-1 receptor homology (TIR) domain superfami
	MsG0580025320.01	1.091	up	7.51×10^{-5}	NB-ARC(IPR002182)丨P-loop containing nucleoside triphosphate hydrolase(IPR027417)丨Leucine-rich repeat domain superfamily(
	MsG0880045625.01	3.454	up	9.92×10^{-5}	Toll/interleukin-1 receptor homology (TIR) domain(IPR000157)丨Toll/interleukin-1 receptor homology (TIR) domain superfami
	MsG0880043897.01	-2.216	down	1.90×10^{-4}	NB-ARC(IPR002182)丨P-loop containing nucleoside triphosphate hydrolase(IPR027417)
	MsG0480023383.01	1.040	up	3.01×10^{-4}	WRKY domain(IPR003657)丨WRKY domain superfamily(IPR036576)
	MsG0480021585.01	1.546	up	3.27×10^{-4}	NB-ARC(IPR002182)丨P-loop containing nucleoside triphosphate hydrolase(IPR027417)

Note: In host–symbiont interaction transcriptomes from nodule tissues, total RNA is typically dominated by host transcripts; therefore, a relatively low proportion of rhizobial reads is a common observation. The mapped rate (%) indicates the proportion of rhizobial signal within total reads and should not be directly compared with the alignment rate of a single-species transcriptome.

Table S7. Differentially expressed genes in key rhizobial functional modules (excerpt). Genes are grouped by functional modules (subtables). Comparison: G9L8 vs WE2. log2FC > 0 indicates upregulation in G9L8.

Table S7A. DEGs related to nitrogen fixation (nif).

Category	GeneID	Name	Product	log_2FC	Direction	FDR
**Nitrogen fixation** (nif)	SM2011_RS02255	nifA	nif-specific transcriptional activator NifA	-1.943	down	6.72×10^{-21}
	SM2011_RS02245	nifT	putative nitrogen fixation protein NifT	-1.825	down	2.73×10^{-18}
	SM2011_RS02250	nifB	nitrogenase cofactor biosynthesis protein NifB	-1.719	down	8.20×10^{-18}
	SM2011_RS02300	nifX	nitrogen fixation protein NifX	-1.614	down	2.33×10^{-16}
	SM2011_RS02295	nifE	nitrogenase iron-molybdenum cofactor biosynthesis protein NifE	-1.472	down	8.39×10^{-15}
	SM2011_RS02290	nifK	nitrogenase molybdenum-iron protein subunit beta	-1.451	down	1.58×10^{-14}
	SM2011_RS02285	nifD	nitrogenase molybdenum-iron protein alpha chain	-1.421	down	2.80×10^{-14}
	SM2011_RS02280	nifH	nitrogenase iron protein	-1.341	down	2.19×10^{-13}
	SM2011_RS02420	nifN	nitrogenase iron-molybdenum cofactor biosynthesis protein NifN	-1.330	down	4.01×10^{-13}

Table S7B. DEGs related to microaerobic respiration/electron transfer (FixA/FixB/FixH/FixQ and cbb3 assembly).

Category	GeneID	Name	Product	log_2FC	Direction	FDR
Fix/FixQ	SM2011_RS03300	SM2011_RS03300	FixH family protein	-1.878	down	2.25×10^{-17}
	SM2011_RS02270	SM2011_RS02270	electron transfer flavoprotein subunit alpha/FixB family protein	-1.634	down	6.94×10^{-17}
	SM2011_RS02275	SM2011_RS02275	electron transfer flavoprotein subunit beta/FixA family protein	-1.539	down	1.47×10^{-15}
	SM2011_RS03315	SM2011_RS03315	CcoQ/FixQ family Cbb3-type cytochrome c oxidase assembly chaperone	-1.570	down	2.14×10^{-12}
	SM2011_RS02100	SM2011_RS02100	CcoQ/FixQ family	-1.556	down	7.25×10^{-11}

	SM2011_RS03340	fixT	Cbb3-type cytochrome c oxidase assembly chaperone transcriptional regulator FixT	-1.232	down	5.91×10^{-9}

Table S7C. DEGs related to nodulation signaling (nod/noe/nol).

Category	GeneID	Name	Product	log₂FC	Direction	FDR
Nodulation signaling （nod/noe/nol）	SM2011_RS02125	noeA	nodulation protein NoeA	-1.468	down	5.50×10^{-14}
	SM2011_RS02130	noeB	nodulation protein NoeB	-1.221	down	3.90×10^{-11}
	SM2011_RS02115	nodL	nodulation O-acetyltransferase NodL	-1.135	down	6.51×10^{-10}
	SM2011_RS02395	nodI	nodulation factor ABC transporter ATP-binding protein NodI	2.686	up	1.51×10^{-4}
	SM2011_RS02405	nodB	chitooligosaccharide deacetylase NodB	2.614	up	2.69×10^{-3}
	SM2011_RS02425	nodN	nodulation protein NodN	13.304	up	6.51×10^{-3}
	SM2011_RS02435	nolF	nodulation protein NolF	1.804	up	1.38×10^{-2}

Table S7D. DEGs related to chemotaxis/motility/flagella.

Category	GeneID	Name	Product	log₂FC	Direction	FDR
Upregulated chemotaxis/motility/flagella	SM2011_RS10340	SM2011_RS10340	methyl-accepting chemotaxis protein	2.428	up	1.70×10^{-6}
	SM2011_RS2650	fliI	flagellar protein export ATPase FliI	2.506	up	2.98×10^{-6}
	SM2011_RS2620	fliG	flagellar motor switch protein FliG	2.740	up	8.71×10^{-6}
	SM2011_RS15015	SM2011_RS15015	pilus assembly protein	2.919	up	1.25×10^{-5}
	SM2011_RS2560	SM2011_RS2560	chemotaxis protein CheA	2.142	up	2.03×10^{-5}
	SM2011_RS2740	motC	chemotaxis protein MotC	2.252	up	4.70×10^{-5}
	SM2011_RS2800	fliR	flagellar biosynthetic protein FliR	2.573	up	4.93×10^{-5}
	SM2011_RS15050	SM2011_RS15050	pilus assembly protein CpaD	2.983	up	5.62×10^{-5}
	SM2011_RS8805	SM2011_RS8805	TadG family pilus assembly protein	2.121	up	6.72×10^{-5}
	SM2011_RS2630	SM2011_RS2630	flagellar motor switch	2.455	up	7.65×10^{-5}

protein FliM

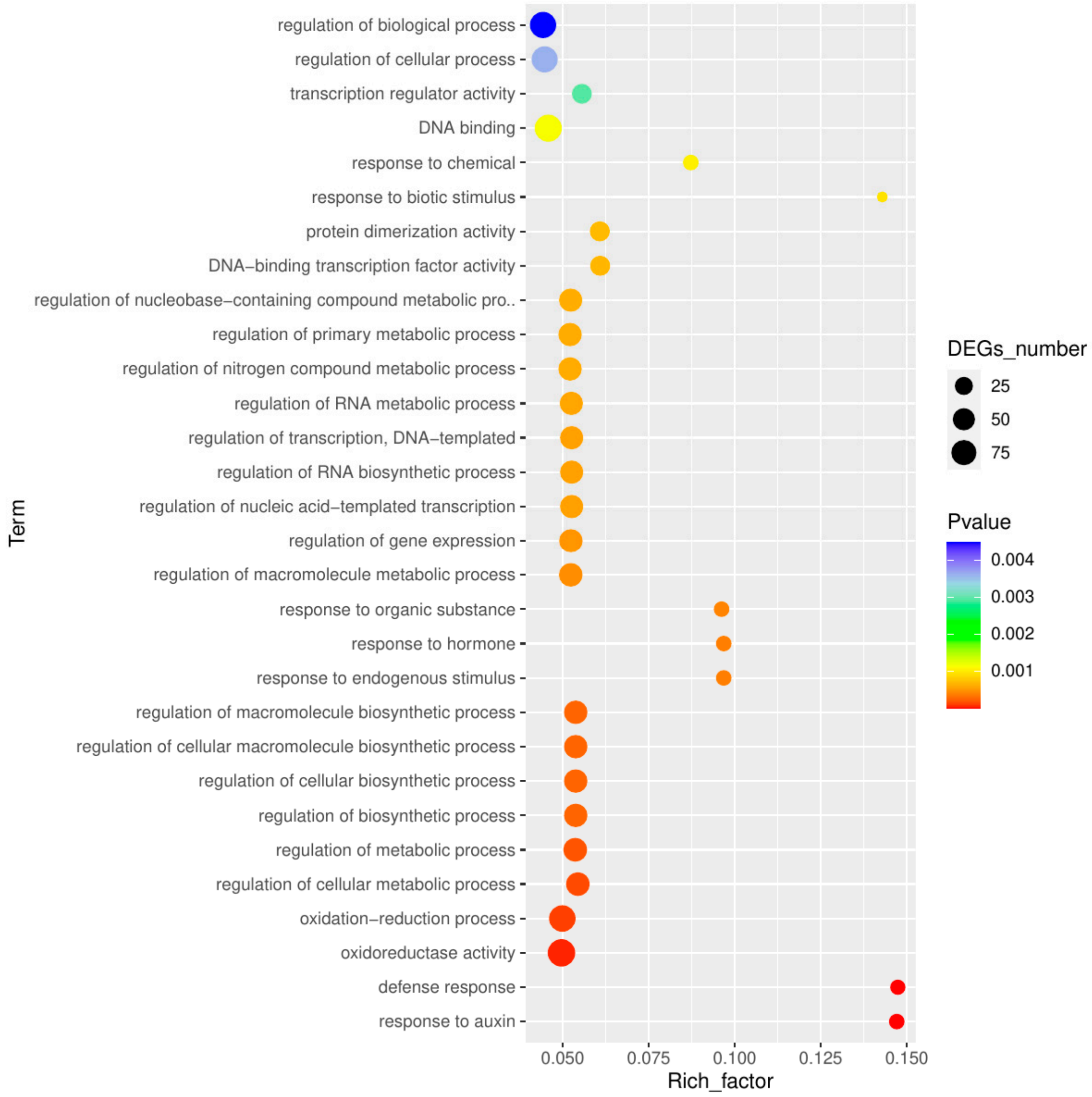

Figure S1. GO enrichment bubble plot for alfalfa DEGs (overall). The x-axis indicates the rich factor; bubble size represents the number of DEGs; color indicates the P value.

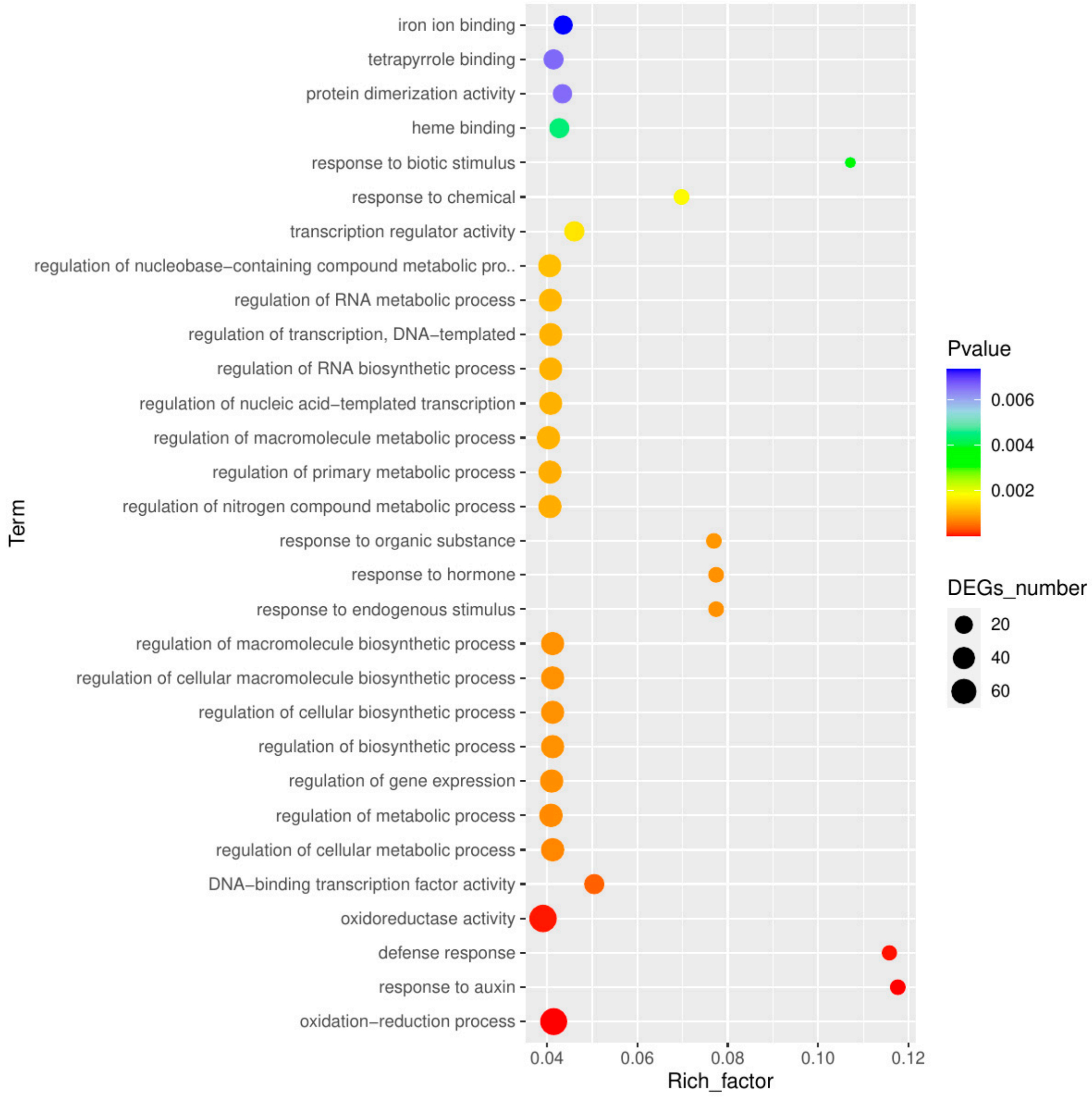

Figure S2. GO enrichment bubble plot for alfalfa DEGs (directional subset 1), highlighting significant terms related to transcriptional regulation, hormone responses, and responses to biological stimuli.

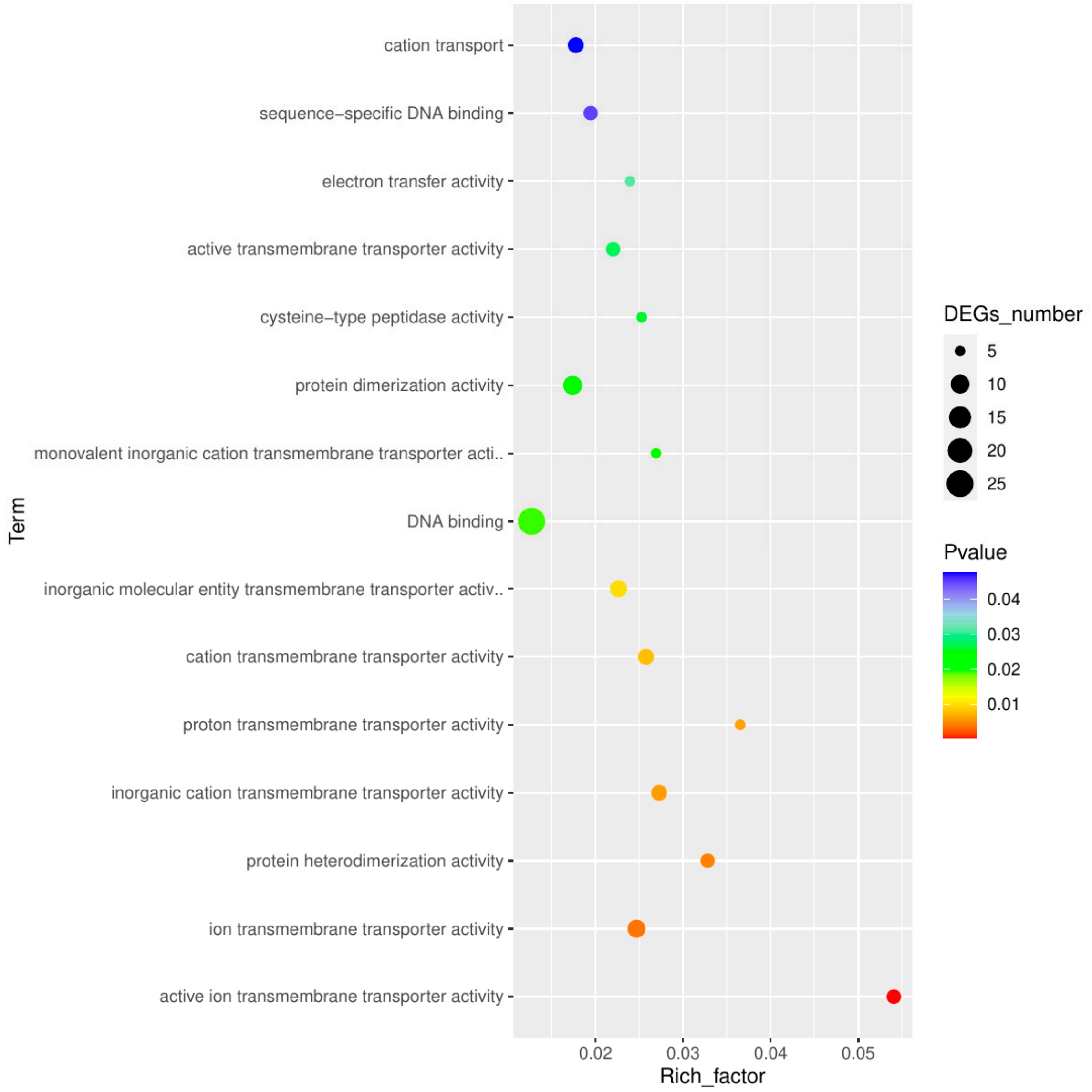

Figure S3. GO enrichment bubble plot for alfalfa DEGs (directional subset 2), enriched in terms related to transmembrane transport, ion transport, and electron transfer.

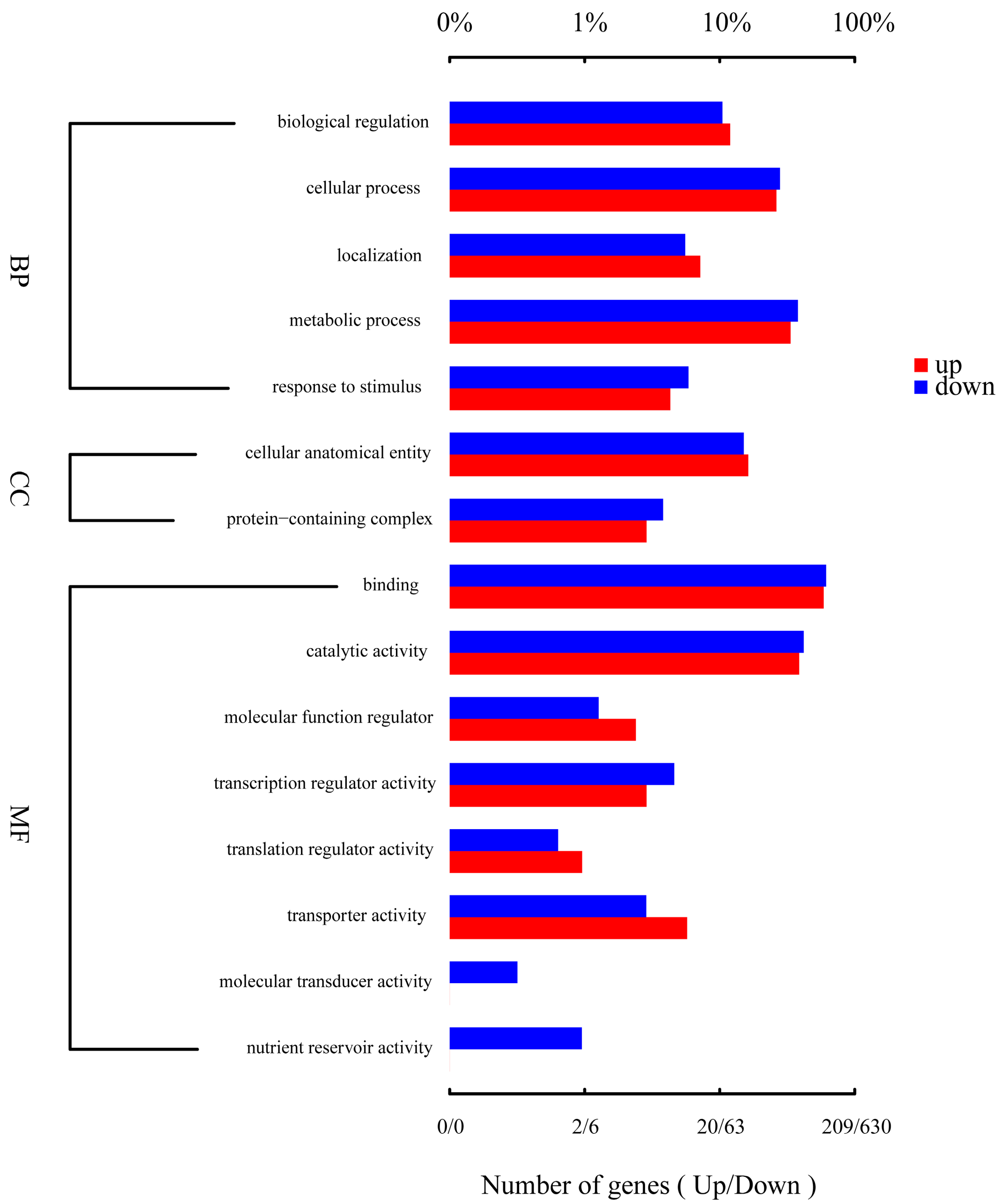

Figure S4. GO functional classification of alfalfa DEGs. Numbers of up- and down-regulated genes are shown across the three GO domains (BP, biological process; CC, cellular component; MF, molecular function).

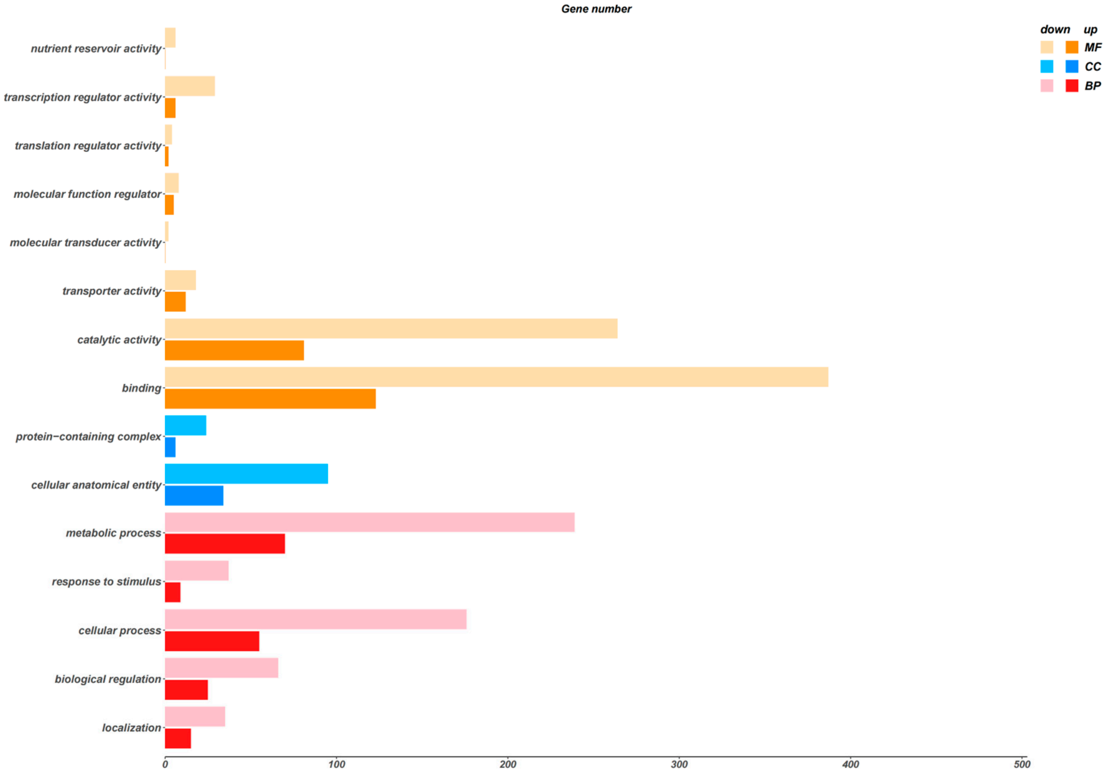

Figure S5. Distribution of upregulated and downregulated alfalfa DEGs across BP/CC/MF categories (shown on a logarithmic scale).

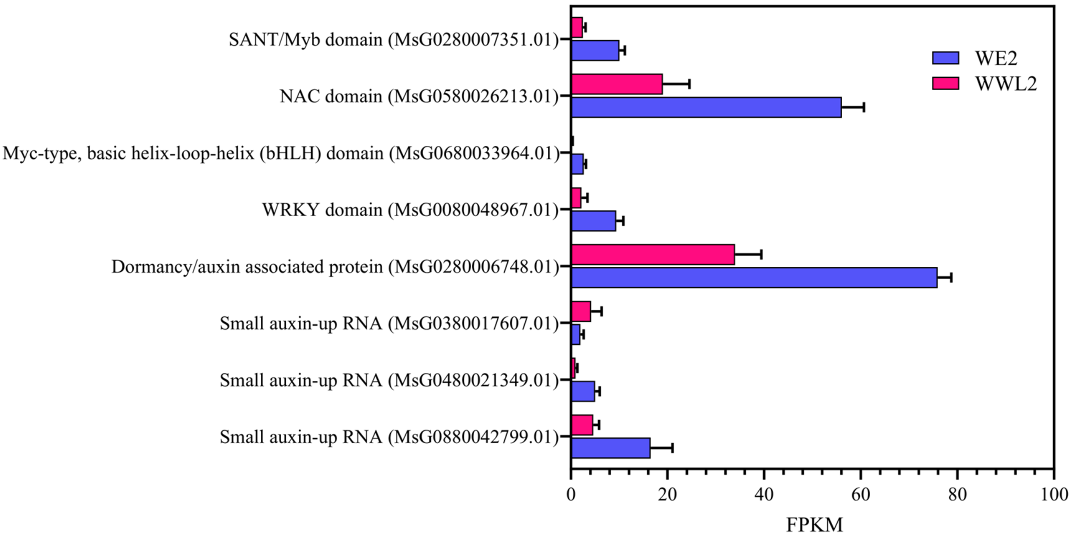

Figure S6. Hormones and transcriptional regulation (auxin and key signaling/transcription factors)(N=3)

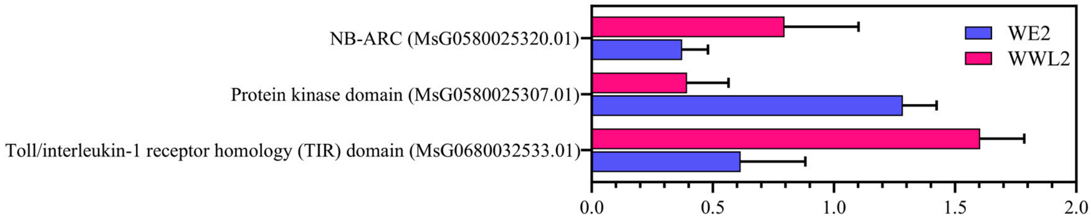

Figure S7. Immune recognition-related genes (receptor-like proteins and defense regulation)(N=3)

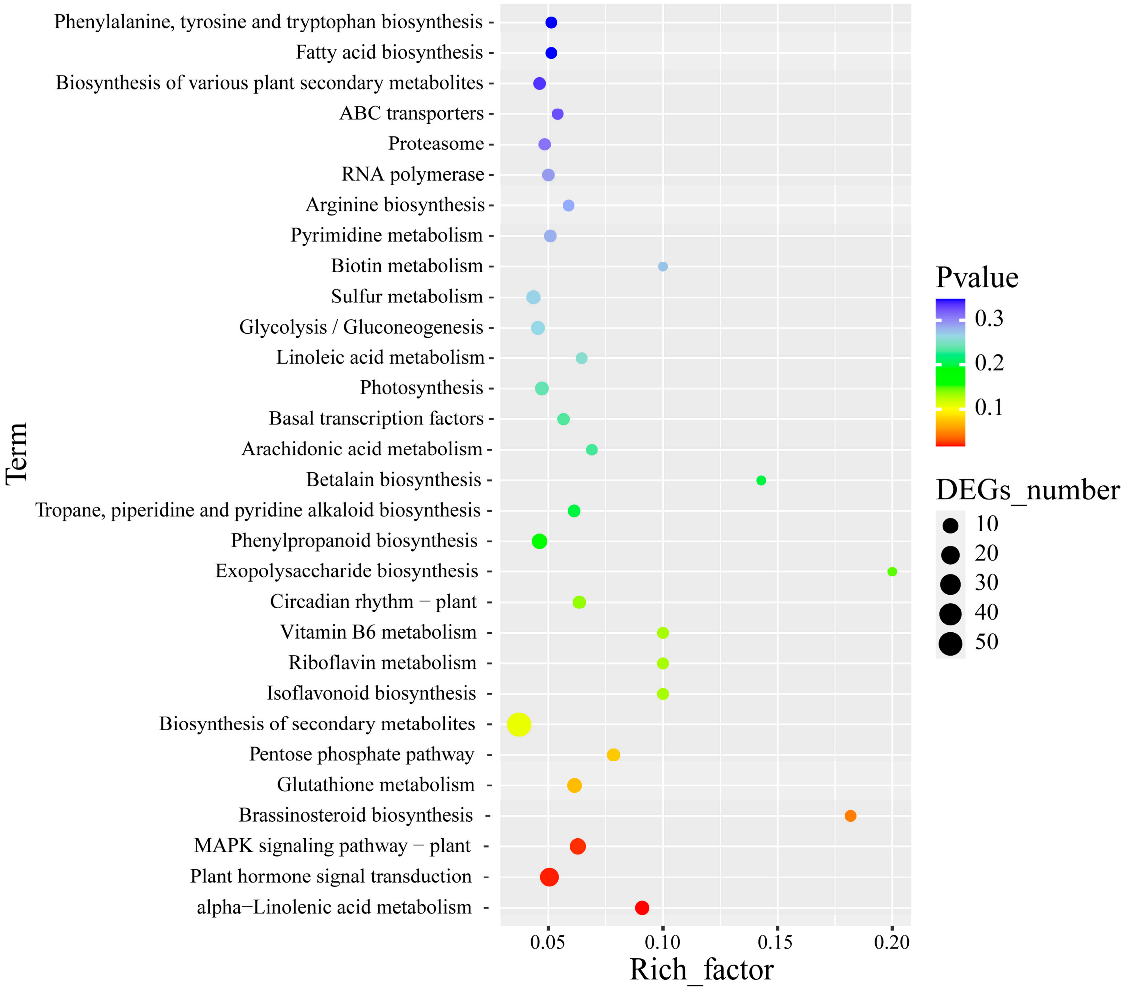

Figure S8. KEGG enrichment bubble plot for alfalfa DEGs (overall). Hormone signaling, MAPK signaling, and α-linolenic acid metabolism show significant enrichment.

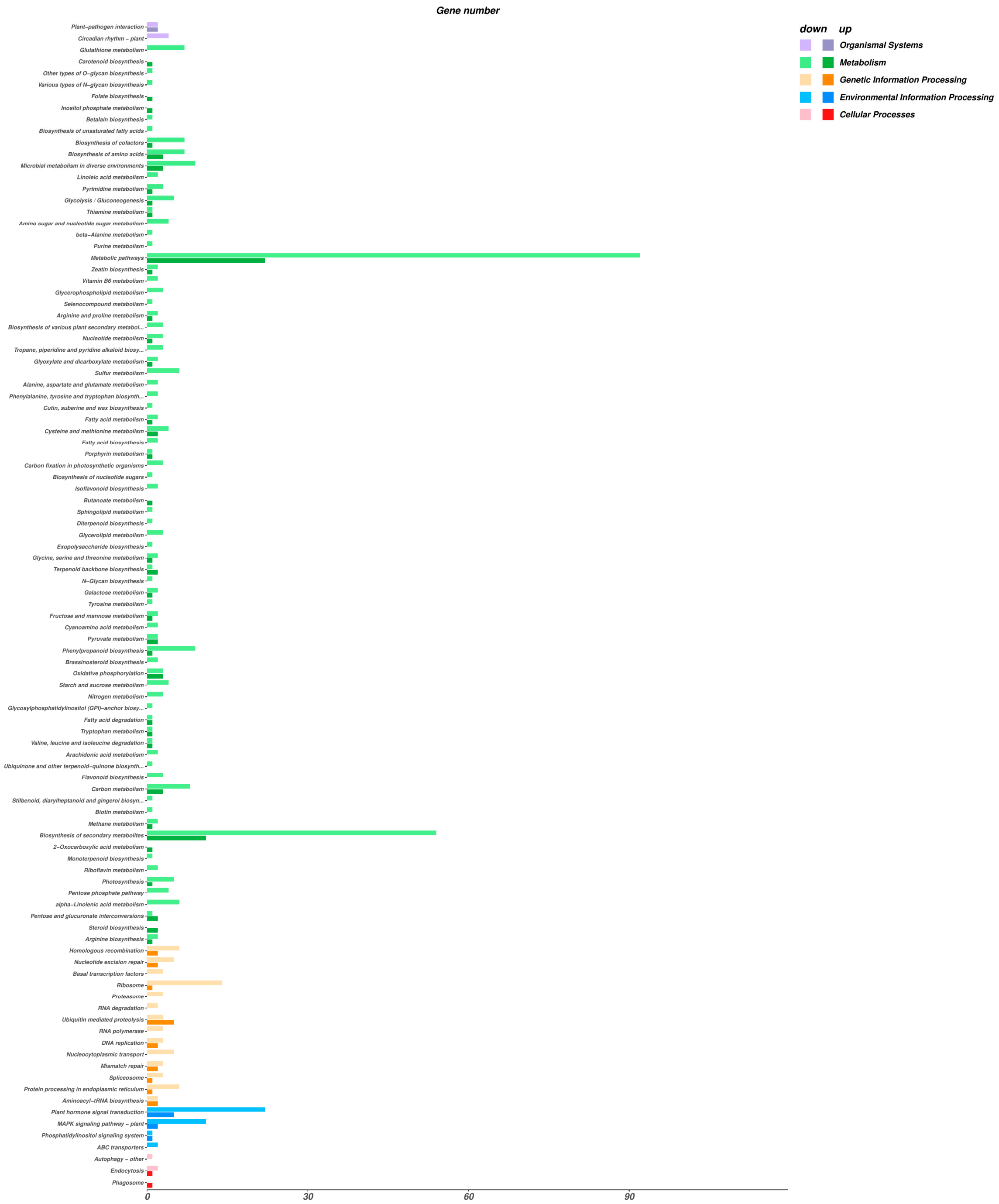

Figure S9. KEGG pathway classification statistics for alfalfa DEGs (counts shown separately for upregulated and downregulated genes).

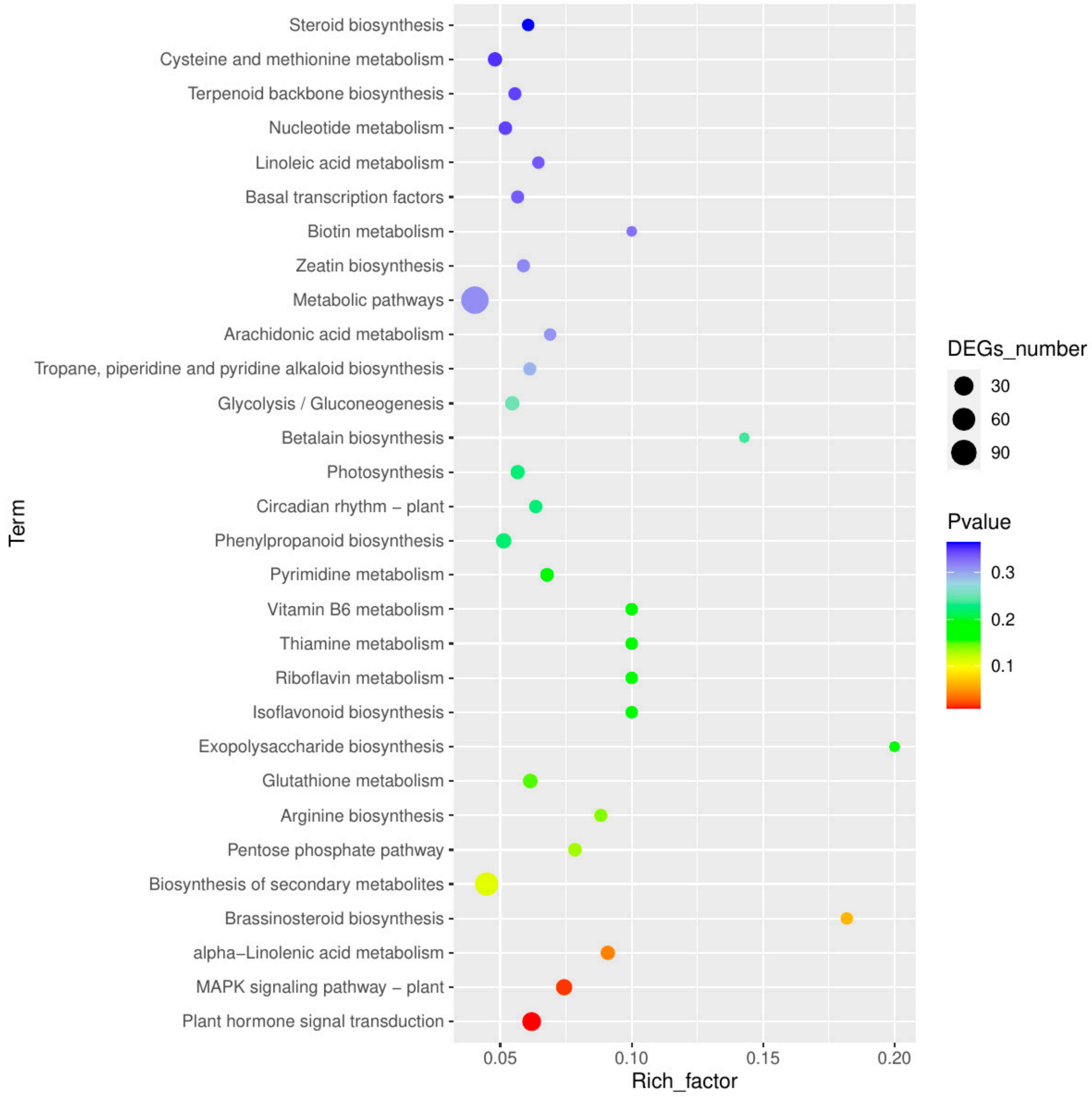

Figure S10. KEGG enrichment bubble plot for alfalfa DEGs (directional subset 1).

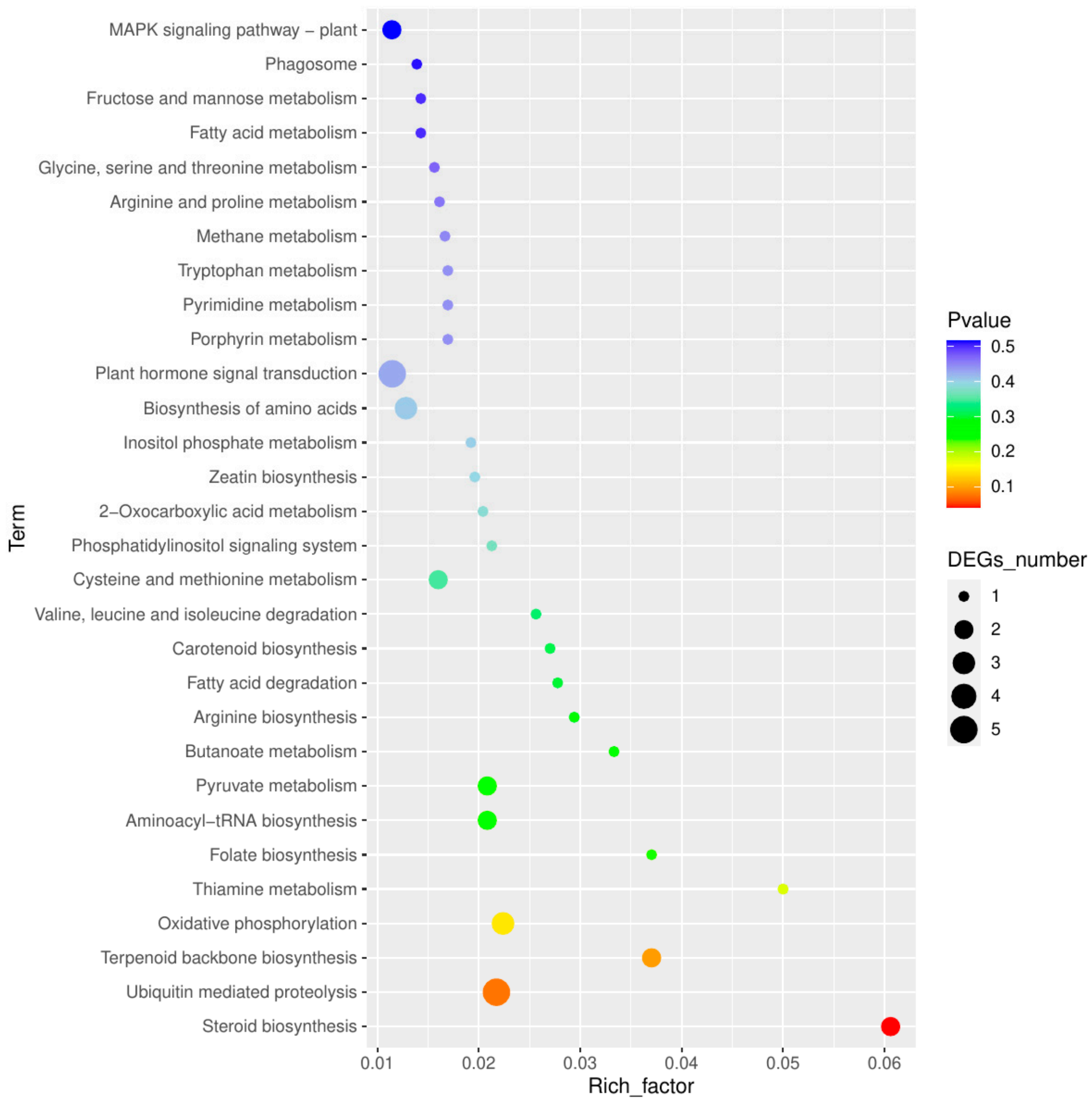

Figure S11. KEGG enrichment bubble plot for alfalfa DEGs (directional subset 2), including steroid biosynthesis, ubiquitin-mediated proteolysis, oxidative phosphorylation, and related pathways.

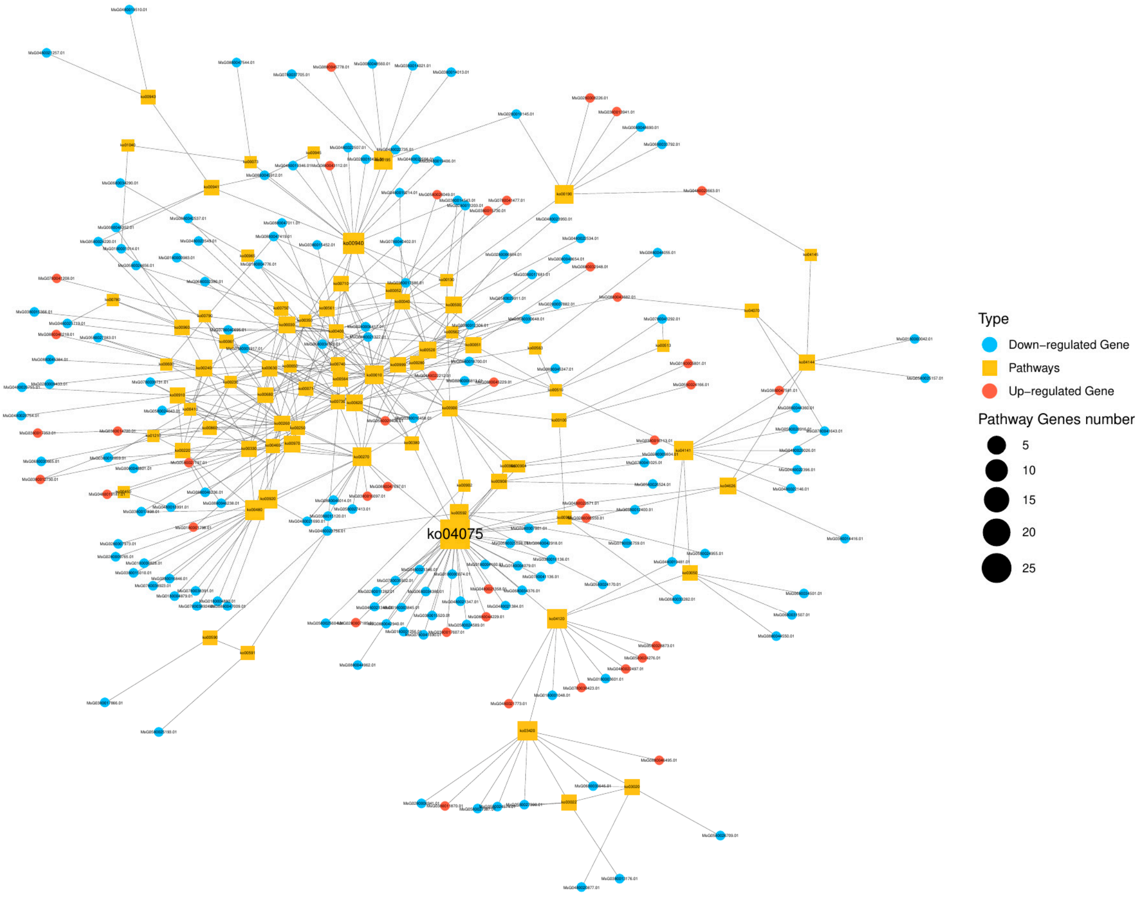

Figure S12. KEGG pathway–gene association network for alfalfa DEGs. Squares represent pathways and circles represent genes; colors distinguish upregulated and downregulated genes.

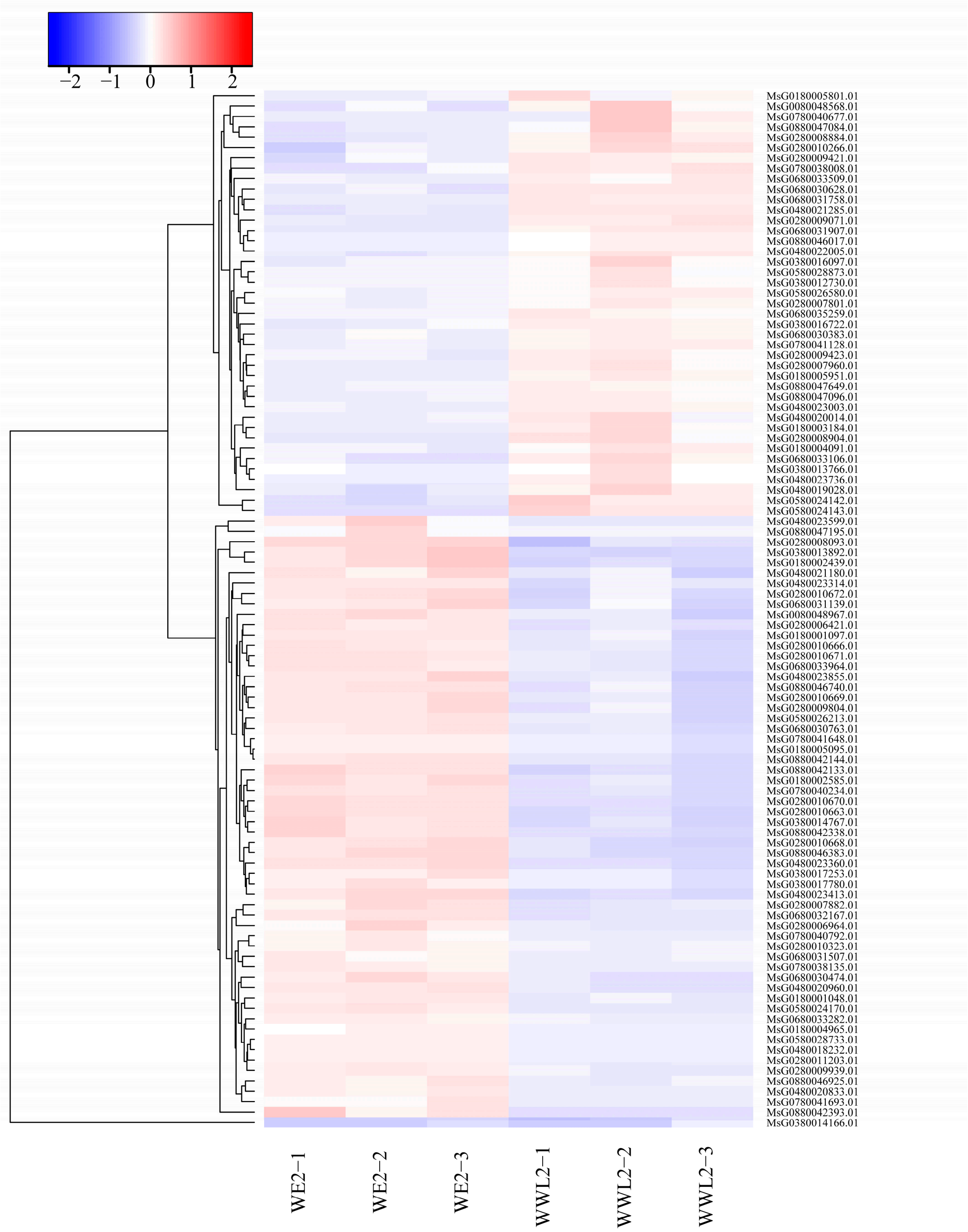

Figure S13. Clustering heat map of alfalfa DEGs.

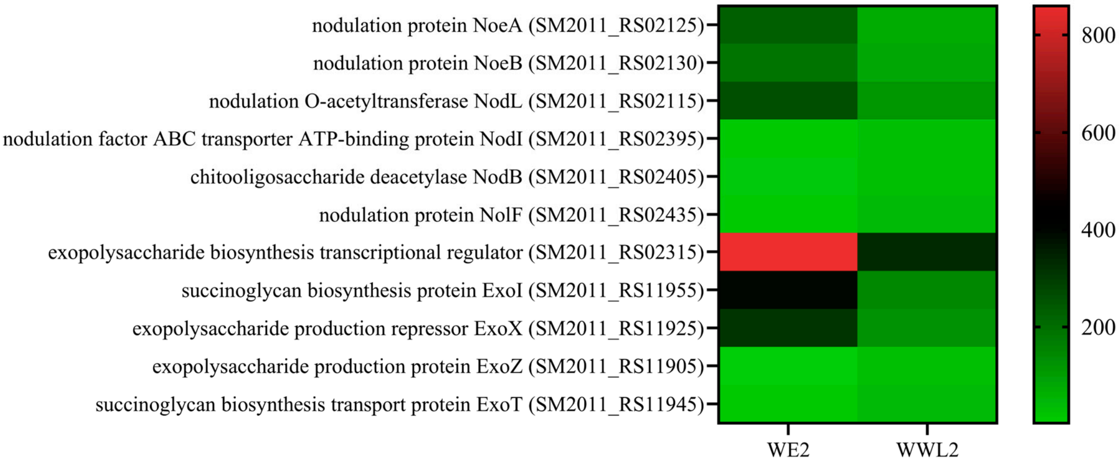

Figure S14. Nodulation signals and surface structures (nod/noe and exo/syr, etc.) (*WWL2* vs *WE2*).

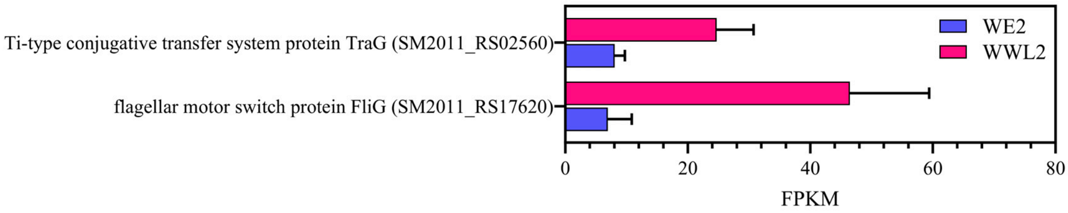

Figure S15. Chemotaxis and motility (che/fli, etc.) (*WWL2* vs ***WE2***).

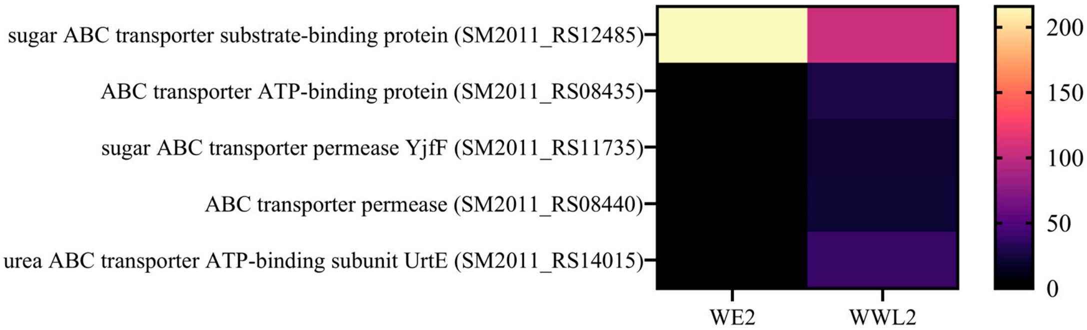

Figure S16. Transport and nutrient acquisition (ABC, etc.) (*WWL2* vs *WE2*).

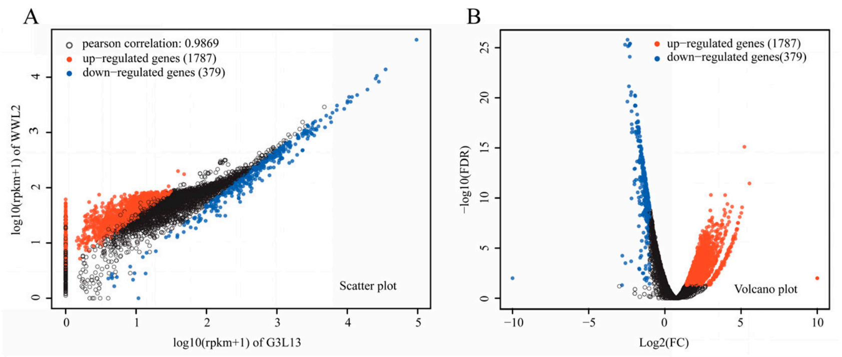

Figure S17. Overview of differential expression on the rhizobial side. (A) Expression scatter plot (log_{10}(RPKM+1)); (B) Volcano plot of DEGs.

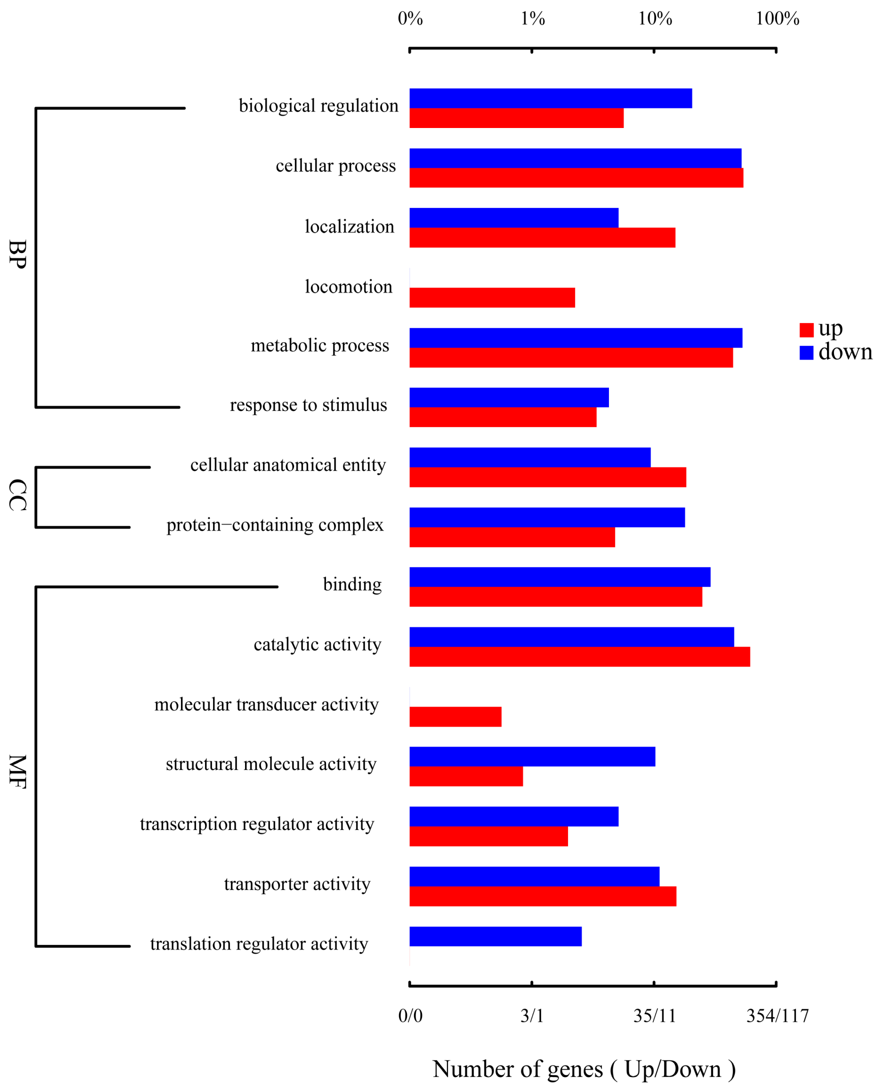

Figure S18. GO functional classification of rhizobial DEGs (BP/CC/MF).

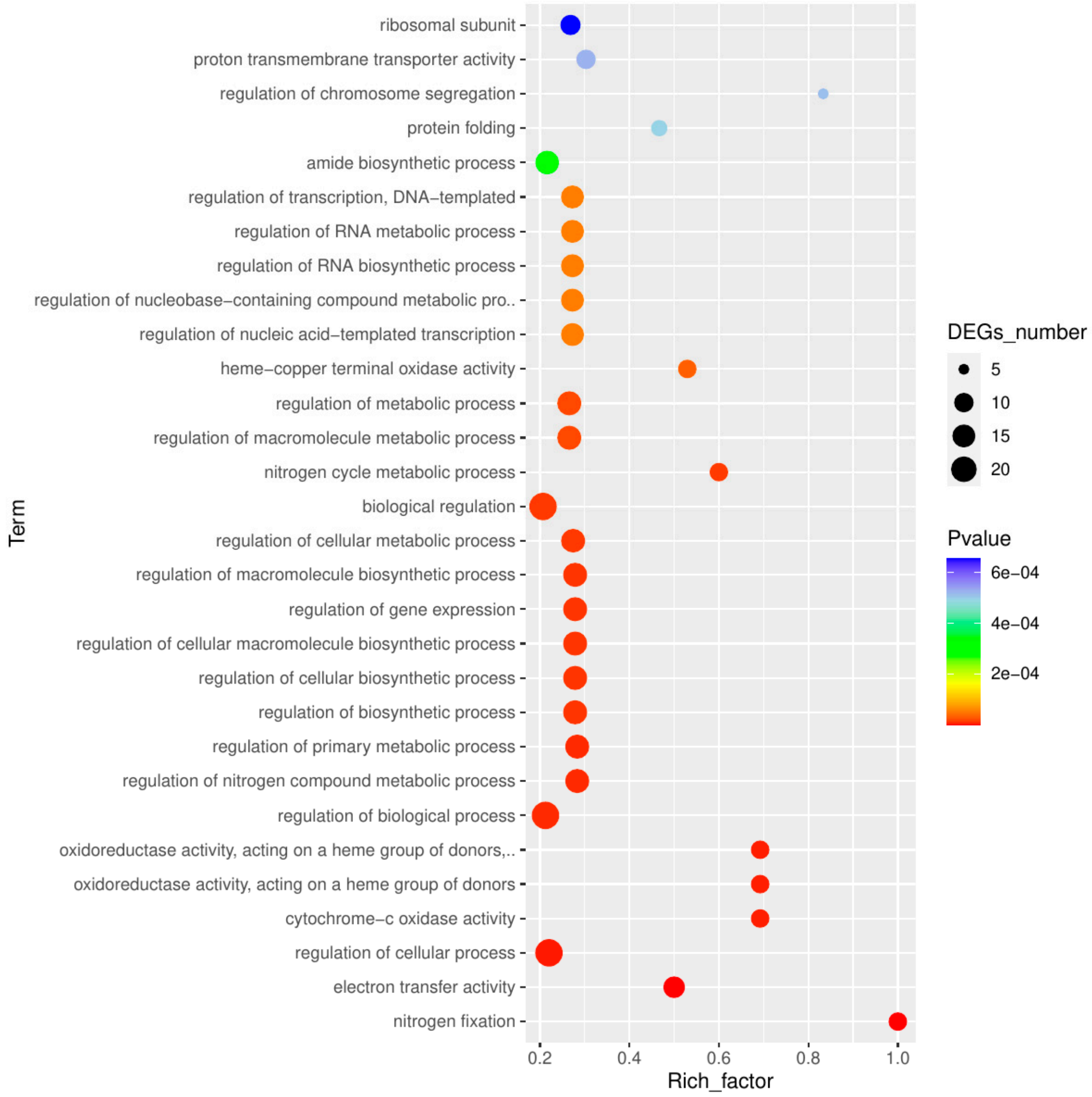

Figure S19. GO enrichment bubble plot for rhizobial DEGs (overall), highlighting terms related to nitrogen fixation, electron transfer, and redox processes.

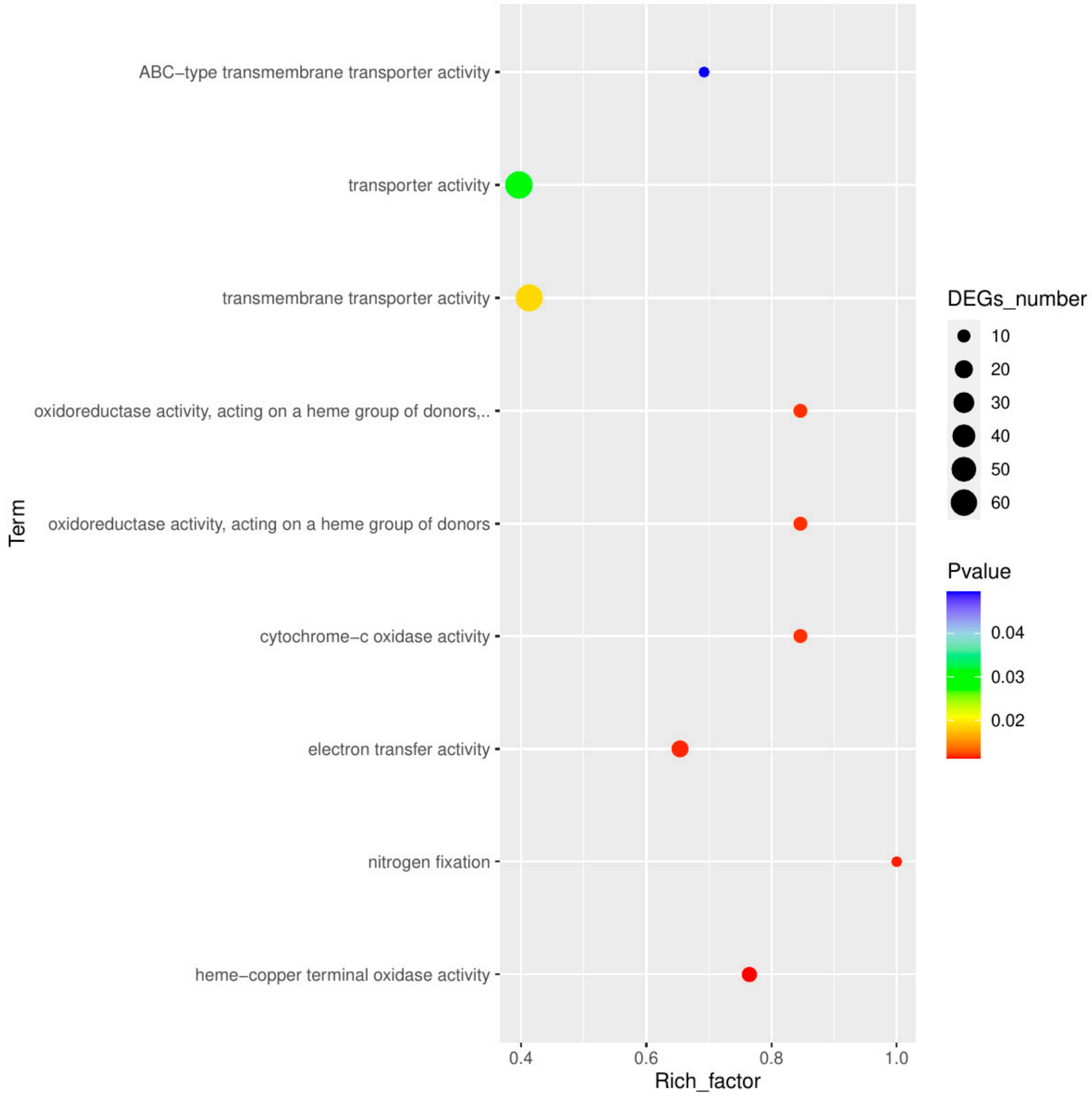

Figure S20. GO enrichment bubble plot for rhizobial DEGs (transport/ion-transport related terms).

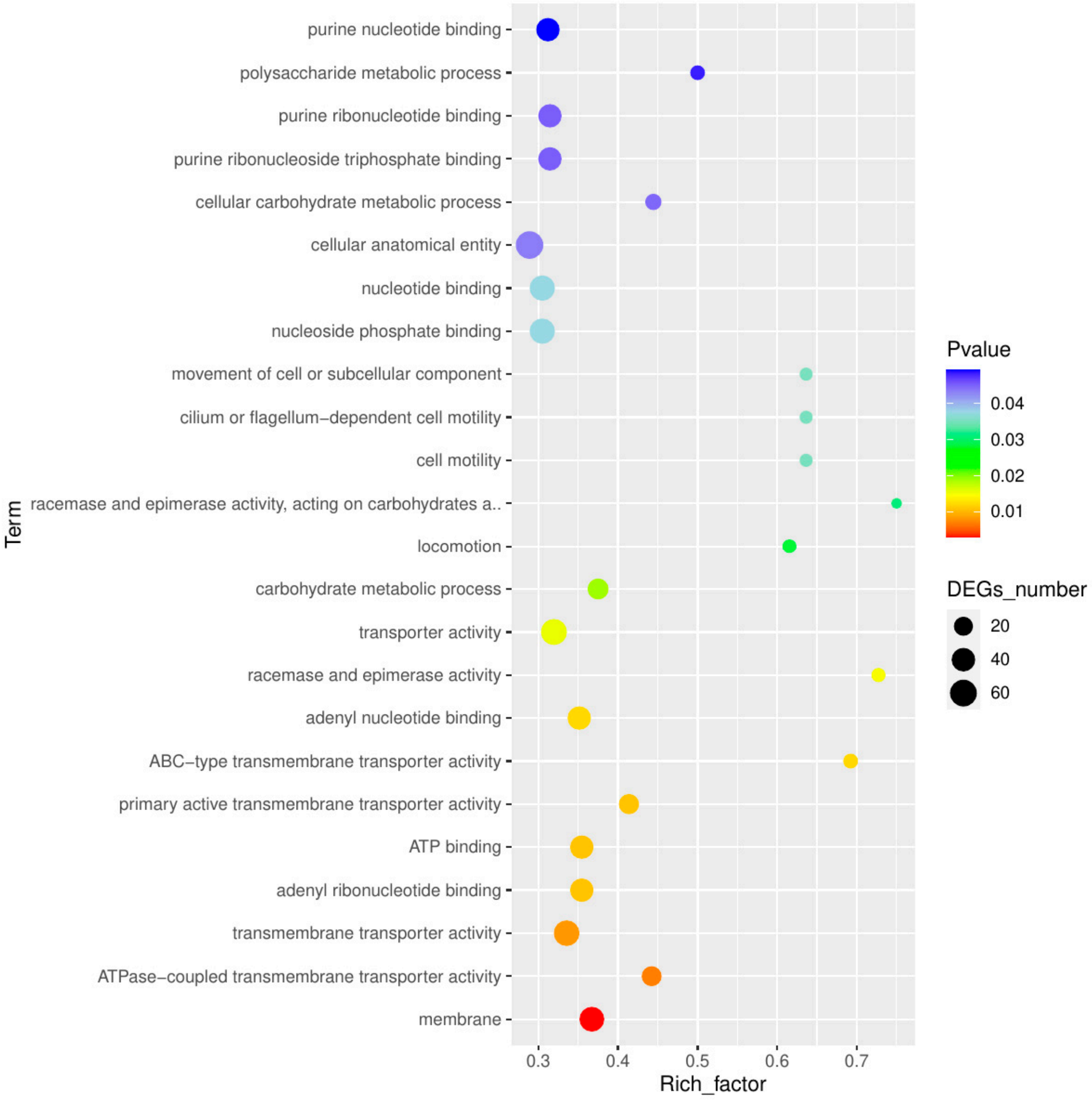

Figure S21. GO enrichment bubble plot for rhizobial DEGs (behavior/stimulus-response related terms).

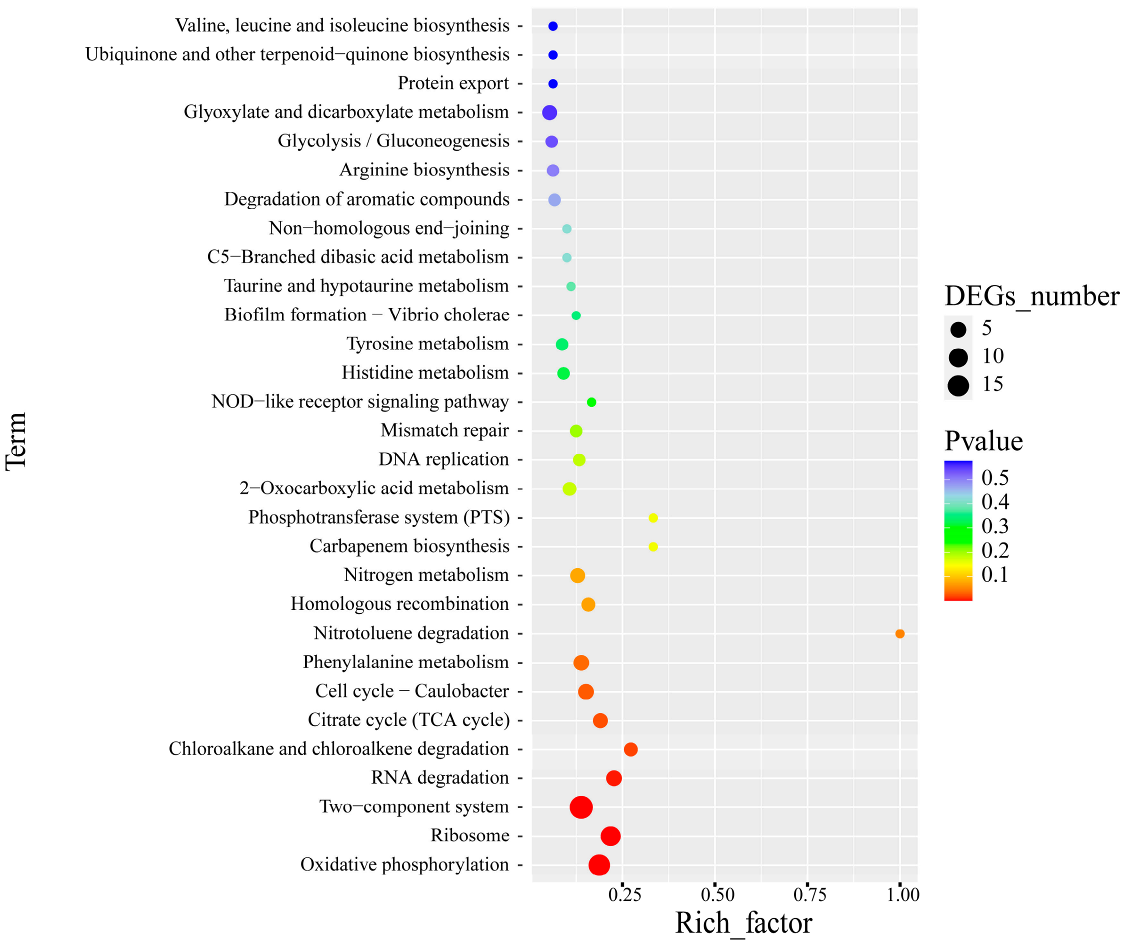

Figure S22. KEGG enrichment bubble plot for rhizobial DEGs (overall). Oxidative phosphorylation and ribosome pathways show strong enrichment.

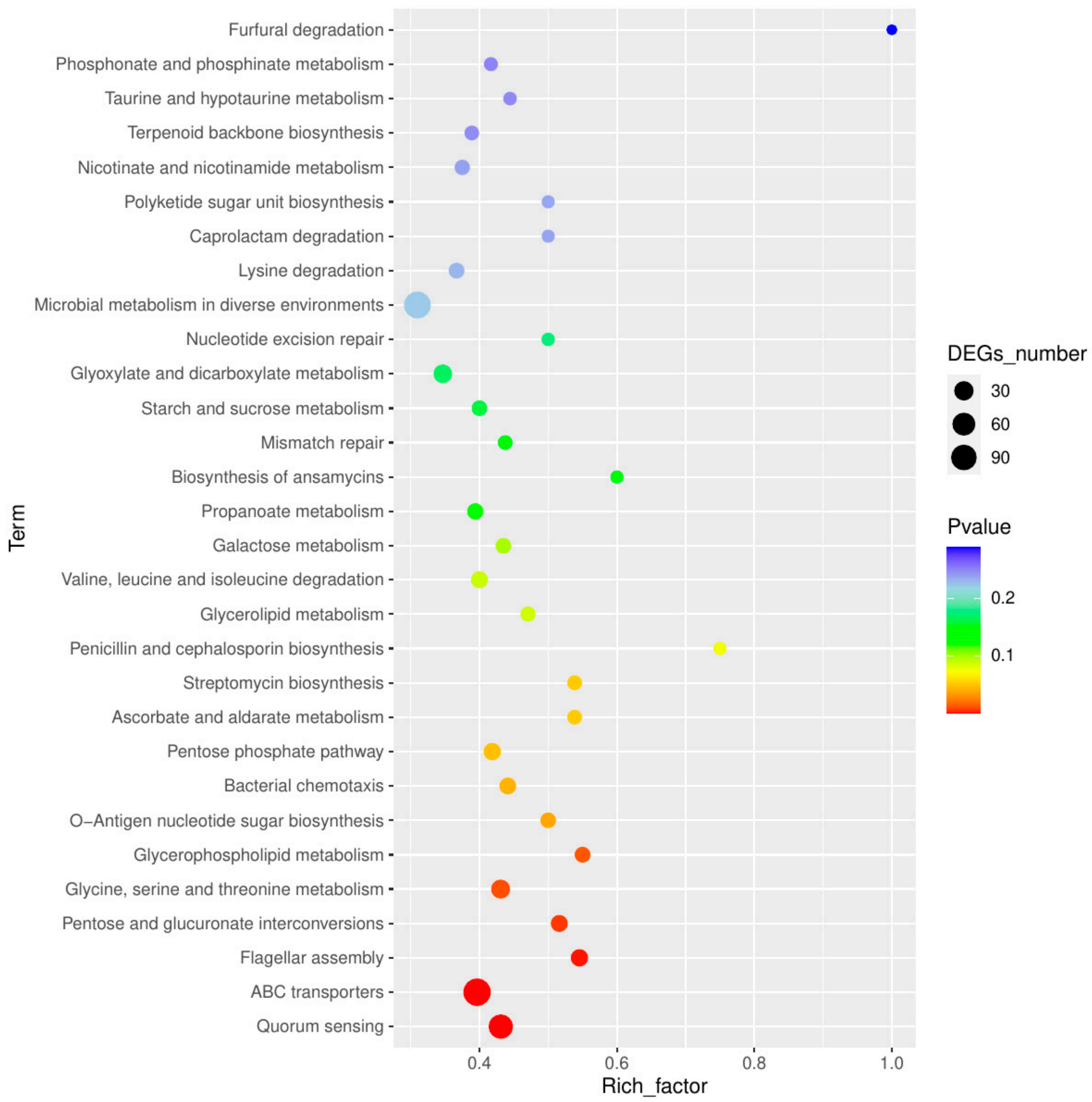

Figure S23. KEGG enrichment bubble plot for rhizobial DEGs (subset 1).

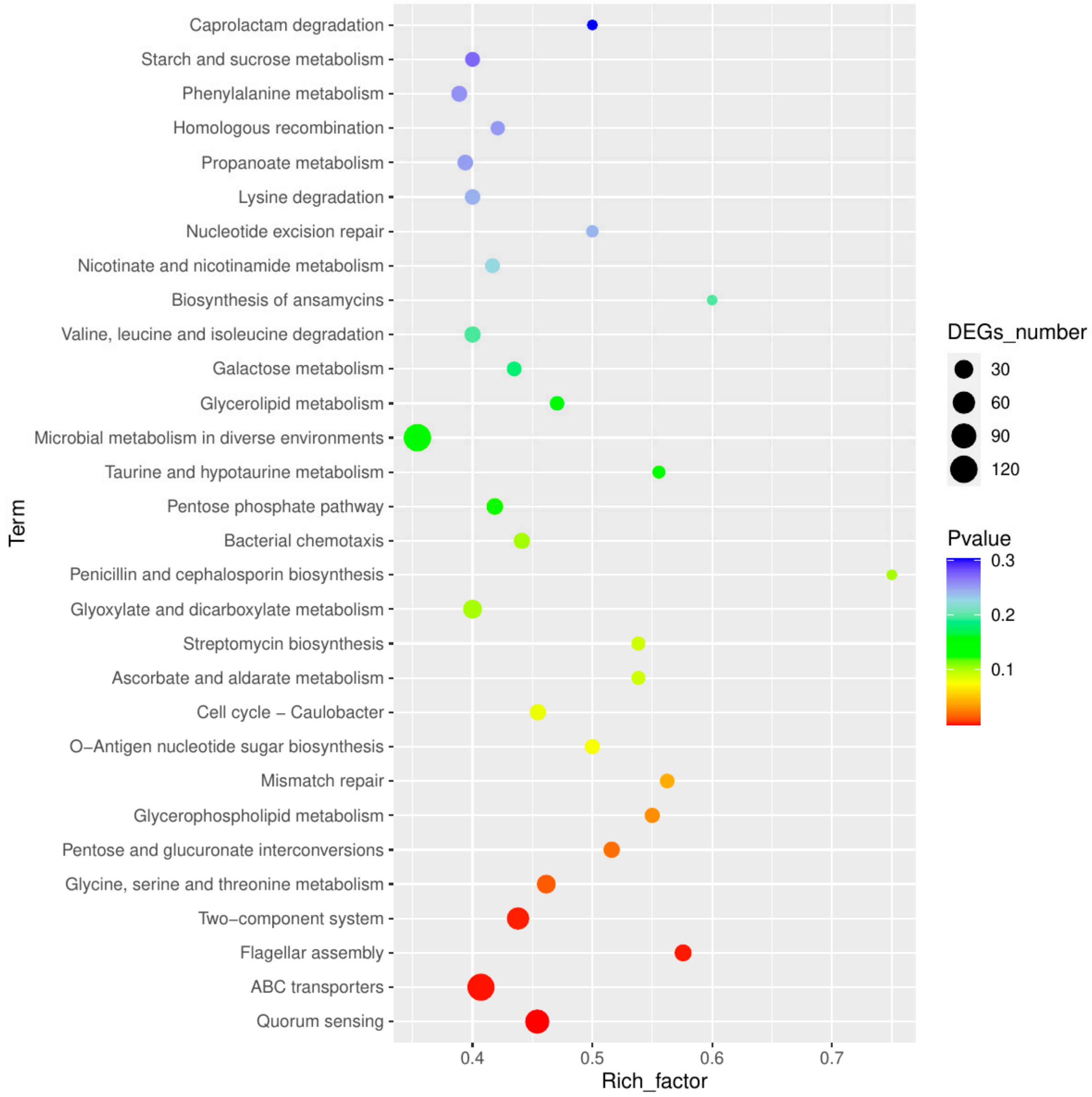

Figure S24. KEGG enrichment bubble plot for rhizobial DEGs (subset 2).

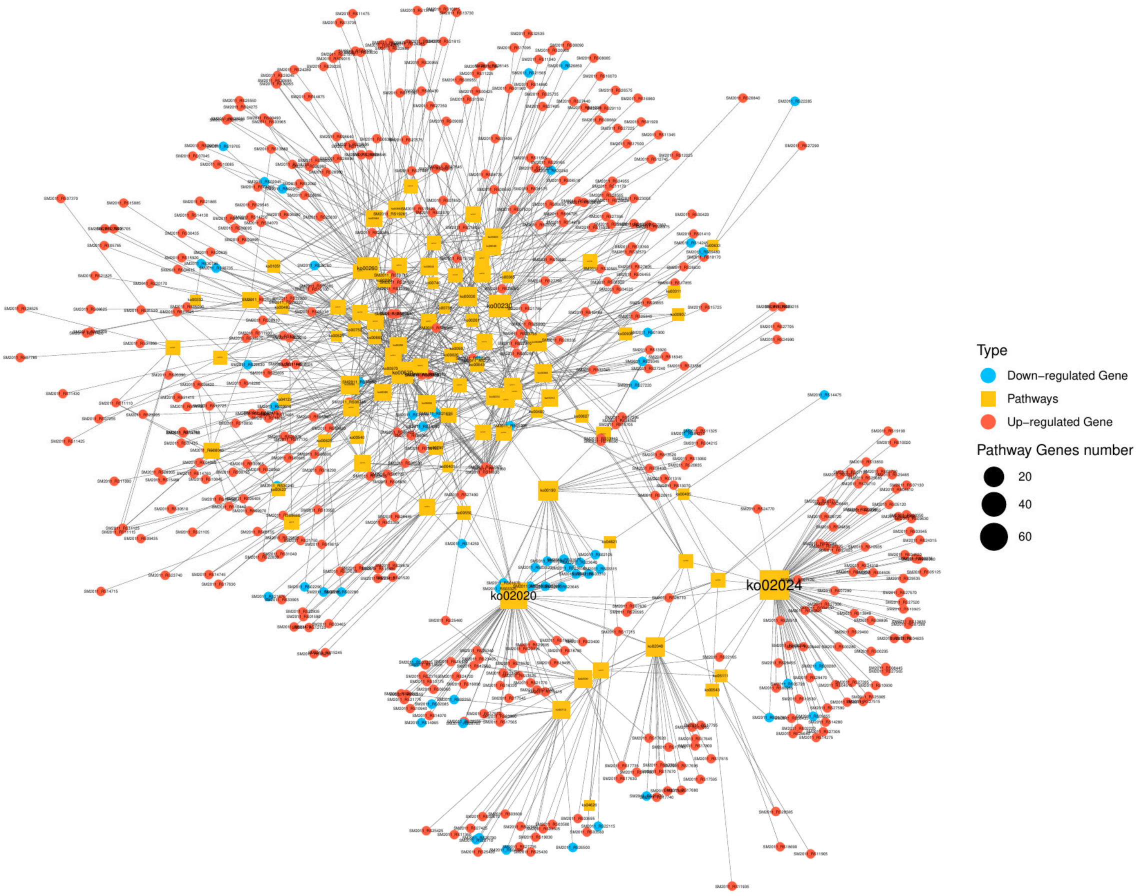

Figure S25. KEGG pathway–gene association network for rhizobial DEGs. Squares represent pathways and circles represent genes; colors distinguish upregulated and downregulated genes.

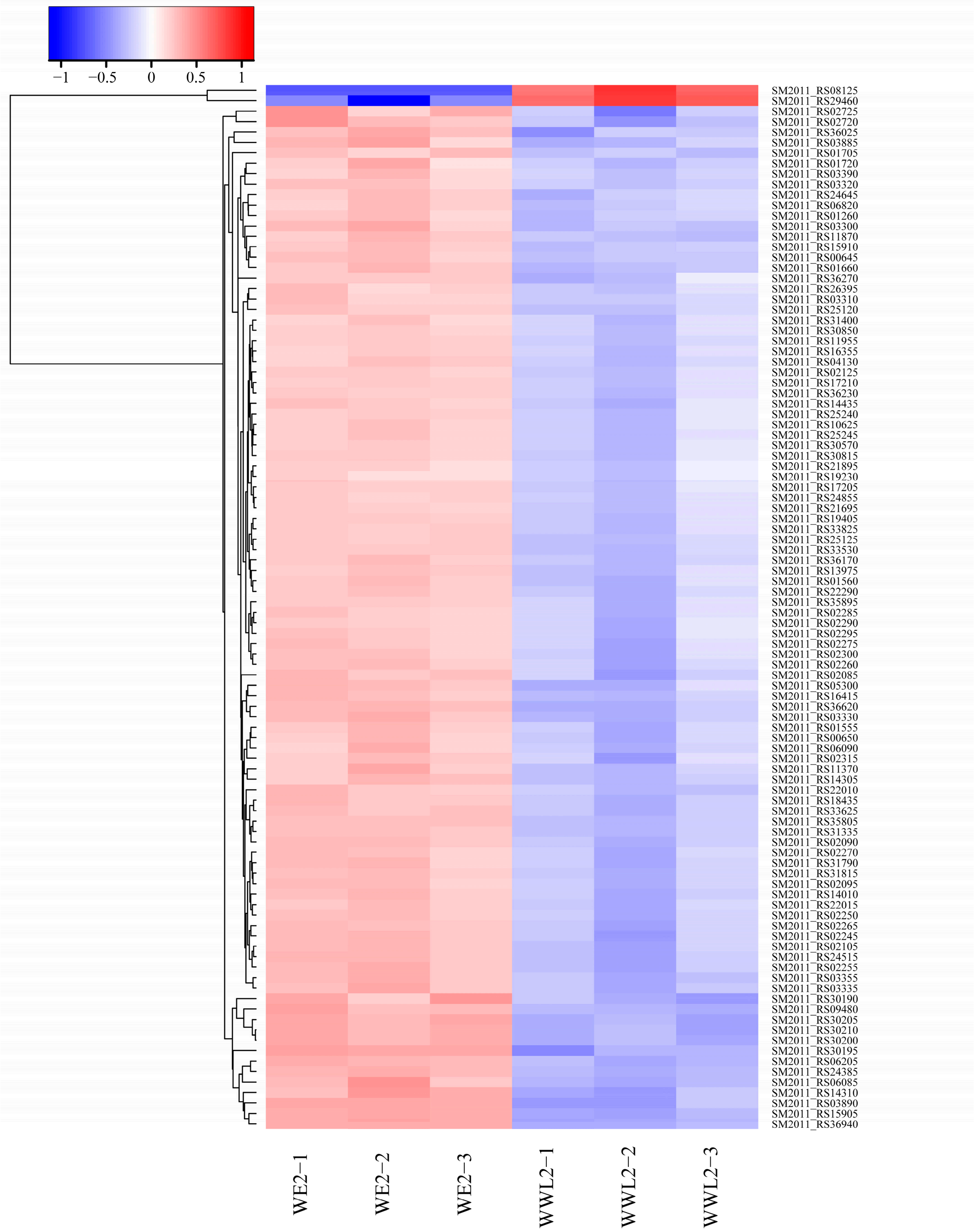

Figure S26. Clustering heat map of rhizobial DEGs.
